# Modeling functional specialization of a cell colony under different fecundity and viability rates and resource constraint

**DOI:** 10.1371/journal.pone.0201446

**Published:** 2018-08-08

**Authors:** Denis Tverskoi, Vladimir Makarenkov, Fuad Aleskerov

**Affiliations:** 1 International Laboratory of Decision Choice and Analysis, National Research University Higher School of Economics (HSE), Moscow, Russian Federation; 2 V.A. Trapeznikov Institute of Control Sciences of Russian Academy of Sciences (ICS RAS), Moscow, Russian Federation; 3 Département d’Informatique, Université du Québec à Montréal, Montréal, Canada; Arizona State University & Santa Fe Institute, UNITED STATES

## Abstract

The emergence of functional specialization is a core problem in biology. In this work we focus on the emergence of reproductive (germ) and vegetative viability-enhancing (soma) cell functions (or germ-soma specialization). We consider a group of cells and assume that they contribute to two different evolutionary tasks, fecundity and viability. The potential of cells to contribute to fitness components is traded off. As embodied in current models, the curvature of the trade-off between fecundity and viability is concave in small-sized organisms and convex in large-sized multicellular organisms. We present a general mathematical model that explores how the division of labor in a cell colony depends on the trade-off curvatures, a resource constraint and different fecundity and viability rates. Moreover, we consider the case of different trade-off functions for different cells. We describe the set of all possible solutions of the formulated mathematical programming problem and show some interesting examples of optimal specialization strategies found for our objective fitness function. Our results suggest that the transition to specialized organisms can be achieved in several ways. The evolution of Volvocalean green algae is considered to illustrate the application of our model. The proposed model can be generalized to address a number of important biological issues, including the evolution of specialized enzymes and the emergence of complex organs.

## Introduction

The division of labor and functional specialization emerge ubiquitously in different biological systems and at different levels of life organization. For instance, the division of labor occurs in simple multicellular individuals [1–2], such that cyanobacteria [3–4], mycobacteria [5], Volvocalean green algae [6–7] and multicellular yeast [8]. Various specialization patterns can be observed in different multicellular organisms. We can underline two major directions here: specialization in distinct somatic functions and germ-soma specialization [5–7]. In this work we mainly focus on the emergence of germ-soma specialization.

There are some mathematical models that try to describe the evolution of specialization among somatic functions [5,9]. For example, Ispolatov et al. have considered the process of formation of two-cell aggregates [5]. Each aggregate can exist either in a unicellular or in a two-cell form. The fraction of time that a cell spends in a two-cell form is controlled by cell stickiness, which can evolve in time. Also, each cell produces two metabolites. In a two-cell form cells can exchange the produced metabolites with other cells, whereas a single cell cannot be involved in such an exchange. Ispolatov et al. [5] have shown that multicellular organisms can emerge from genetically identical ancestors and that the benefits of aggregation, achieved through specialization in metabolites production, stimulate this emergence. This aggregation allows increasing the dimension of phenotype space and provides new global maxima of the fitness function. It is worth noting that the changes in cell stickiness can lead to further differentiation of cell types in the colony.

Now we will discuss the main issue of our study: the emergence of germ-soma specialization [10–12]. Volvocalean green algae are the most appropriate biological system for studying this issue [13–14]. Volvocalean green algae are flagellated photosynthetic organisms. Their lineage contains unicellular organisms, multicellular organisms without cell differentiation, multicellular organisms with partial specialization and multicellular organisms with full germ-soma specialization [13]. In their seminal work, Michod et al. [14] have studied the origin of specialization in colonies of identical cells. The fitness of the colony has been defined through its two basic components: viability and fecundity. These authors have introduced a specific trade-off function reflecting the intrinsic relationships that link viability and fecundity within a given cell. This trade-off emerges due to the cells physiology and other constraints. Michod et al. [14] have shown how the colony’s fitness can be defined using the trade-off functions of individual cells. Their work suggests that the curvature of trade-off functions is an important factor that influences the emergence of functional specialization. Moreover, Michod et al. [14] have stated that small-sized colonies with low initial costs of reproduction have concave trade-off functions at each cell; large-sized colonies require high initial costs of reproduction and, hence, convex trade-off functions.

Solari et al. [15] have supported the idea that initial costs of reproduction play a significant role in the process of germ-soma separation. The model proposed by these authors allows explaining the GS (undifferentiated colonies)–GS/S (colonies composed of specialized somatic cells and unspecialized cells)–G/S (colonies with complete germ-soma specialization) form of the complexity evolving process in Volvocalean green algae. Hallmann [16] has examined in detail the evolution of reproductive development in Volvocalean green algae. Gavrilets [17] has studied the emergence of germ-soma specialization via developmental plasticity. This author has investigated how regulatory gene expressions, mutation rate, size of a colony and costs of plasticity influence the dynamics of the division of labor. Willensdorfer [18] has discussed the phenomenon of somatic cells. No any full-terminate somatic cell can reproduce. It dies after the colony reproduces. This means that these cells should provide some benefits to the organism in order to justify their existence. Willensdorfer has presented a model that allows one to determine whether somatic cells are advantageous for the organism or not, and to calculate the optimal fraction of these cells. Solari et al. [19] have considered the problem under study using some knowledge about different physical processes underlying the organism's evolution. Rueffler et al. [20] have introduced a general mathematical model of multicellular individuals. The main assumption of the model presented in [20] is the presence of some modules that can contribute to different evolutionary functions connected by a trade-off relationship. It has been shown that three factors favor different contributions of modules to tasks–positional effects, accelerating performance functions and interaction between modules. Despite of its generality, the model of Rueffler et al. does not predict whether specialization emerges in the system or not.

Several authors have developed new models based on the concept of a fitness function. In [21–23], the models of social choice have been applied to the problem under study. It has been shown that the use of the axiomatic approach allows one to construct different social welfare functions that describe the types of fitness-ranking on the set of alternatives representing all states relevant to the group. Bossert et al. [23] have suggested applying extensive social welfare functions and using their axiomatic properties to better describe the fitness functions of colonies. These authors have shown that their axioms are in complete agreement with the fact that the emergence of germ-soma specialization is accompanied by replacing concavity by convexity in the trade-off functions.

Most of the fundamental studies discussed above focus on a trade-off relationship between the germ and somatic functions and, in particular, on how this relationship drives the emergence of specialization. These studies shed light on how different model's parameters, such as the number of cells in the colony, different levels of regulatory genes expression or the initial cost of reproduction, can change the shape of a trade-off relationship. There are, however, some other factors whose impact on the germ-soma separation has not been studied in detail. For instance, it is well known that the emergence of specialization is closely related to environmental conditions [24]. More precisely, a number of empirical studies have shown that the reproduction strategy varies in response to environmental changes [25]. Kisdi et al. [24] have suggested that environmental conditions influence reproductive strategy of cell colonies. Also, differences in environmental factors influence viability of the colony. If we use a trait-like flagellar motility as an approximation of viability, then viability would depend on the amount of resources dissolved in the surrounding fluid [19] and the type of environment, e.g., still or mixed (see [26]). In [27], the rate of survival is used as an approximation of viability. In this case, restricted resources lead to a competition between colonies and this competition influences the rates of survival of all interacting colonies. It means that there exists a relationship linking fecundity of the colony to its viability and environmental factors.

The models described in [14–15] assume that all cells of the colony are identical and thus have the same trade-off relationships. However, this is a very strong simplification. There are several factors which can lead to the emergence of non-identical cells in the colony and to a non-equivalence of cells with respect to biological functions. The first such a factor is the presence of mutations. Mutations and the natural selection trigger and drive the process of evolution. A number of important theoretical approaches to evolution are based on the process of selection involving mutations. One of them uses the concept of adaptive dynamics, which is derived based on the selection gradients [28–30]. Mutations occur during cell divisions and change intrinsic structures of cells. In other words, this means that different cells can have different trade-off functions. Even though initially all cells in the colony are identical, the situation can changes further. For instance, Ispolatov et al. [5] have considered a model where the genetic parameter of stickiness evolves in time and thus takes different values for different cells. A difference between stickiness among cells within the group can lead to the emergence of non-equivalence of cells with respect to a task and, thus, to different trade-off functions of cells.

The second factor here is the presence of a specific developmental program which is characterized by asymmetric divisions [31] (Developmental program 2 in Volvocalean green algae [13]). The activation of the *gls* genes in the embryos leads to the appearance of large-small sister-cell pairs [31]. Gavrilets [17] has provided a model with two prototypes (proto-soma and proto-germ) that can be easily linked to large-small sister-cell pairs. However, it would be also interesting to elucidate how the number of these prototypes evolves and to develop a model that deals with an arbitrary number of prototypes.

The third factor here is the presence of positional effects described by Rueffler et al. [20]. More precisely, positional effects mean that the contribution of different cells to different tasks depends on the position of each cell within the colony. For example, in Volvocalean green algae, cells in the interior or on the edges of the colony do not need to have the same opportunity to contribute to viability. Michod et al. [14] have assumed that specialization occurs first, and pointed out that specialized germ cells are nonflagellated and thus do not contribute to motility. These cells are located in the interior of the colony, making the colony spheroid smaller and lowering drag. Another vision would be that if for some reason cells are located in the interior of the colony, then it would make sense for them to become specialized in germ. Thus, difference in cells locations generates difference in trade-off relationships among cells.

In this work we consider a colony of cells and try to understand incentives for specialization within this colony depending on a given fitness function, encompassing different fecundity and viability rates, and a specific resource constraint. We describe three new mathematical models which represent some important generalizations and extensions of the core model of Michod et al. [14]. Firstly, the colony of identical cells is studied. We examine whether the changes in available resources and/or fecundity and viability rates can increase the number of specialized cells in small-sized and large-sized colonies. Secondly, the colony of cells of different types is studied. This differentiation of types may occur as a result of gene mutations, an asymmetric division or positional effects. It corresponds to the changes in trade-off functions between viability and fecundity within cells. We investigate whether the differentiation of types can lead to the increase in the number of specialized cells in small-sized and large-sized colonies. Moreover, we explore how the difference in the types of trade-off functions, available resources or fecundity and viability rates influences the optimal number of specialized cells in the colony. Thirdly, we describe a possible generalization of the models considered in the two previous sections. The last section provides further discussion and presents some ideas for future research.

## Materials and methods

### Optimization model for the colony of identical cells

#### The model

Consider a colony of *N* cells, with cells indexed *i* = 1,…,*N*. Each cell can contribute to two components of fitness, viability and fecundity. Let *b*_*i*_ be fecundity of the cell *i* and *v*_*i*_ be viability of this cell. Denote *b =* (*b*_1_,..,*b*_*N*_) and *v =* (*v*_1_,..,*v*_*N*_). Also, we assume that there is an intrinsic relationship between viability and fecundity within each cell, called a trade-off relationship between viability and fecundity [14]. Such a trade-off reflects the inner structure of a cell and can be mathematically represented as

$$v_{i}\leq \varphi (b_{i}),\text{for}\;i=1,\ldots ,N.$$ (1)

The function *φ* is the trade-off function between *b*_*i*_ and *v*_*i*_. It describes the relationship between viability and fecundity for all cells of the colony since in this model we assume that all cells within the colony are identical (i.e., they have the same intrinsic structure).

Here we describe a set of important requirements that the trade-off function *φ* should satisfy. First of all, we define the constant *b*^max^∈*R*^+^, such that b^max^ < ∞. This constant represents the maximal level of fecundity achieved by a cell, which is bounded due to the cell physiology. We assume that the contribution to fecundity provided by each cell cannot be negative. Thus, we can describe the trade-off function as $\varphi :[0,b^{\max}]\to R^{+}\cup \{0\}$. For the sake of simplicity we assume that the function *φ* is continuous on [0,*b*^max^] and twice continuously-differentiable on (0,*b*^max^). The investment in one biological component of fitness (viability or fecundity) detracts from the other, leading to the following property: $\frac{d\varphi }{db}<0$ for all *b*∈(0,*b*^max^). Also, this property implies that *φ*(*b*^max^) = 0, *φ*(0) = *v*^max^, *v*^max^∈*R*^+^ and *v*^max^<∞. This means that if a cell makes the maximal possible contribution to one of the fitness components, its contribution to the other component should be minimal or minimally possible (i.e., zero).

The group’s level of fecundity, *B*, is an additive function of variables *b*_*i*_, *i* = 1,.., *N*. Likewise, the group’s level of viability, *V*, is an additive function of variables *v*_*i*_, *i* = 1,.., *N*:

$$B=\sum_{i=1}^{N}b_{i}\;\text{and}\;V=\sum_{i=1}^{N}v_{i}.$$ (2)

The additive form of the group’s fecundity seems biologically reasonable, but the additive form of the group’s viability is more questionable. Following Michod et al. [14], we use a trait-like flagellar motility as an approximation of viability. Hence, this additive form of the group viability is appropriate here (further, we show that in many cases the assumption of additivity is not necessary for the viability function).

Now we will define the fitness of the colony. The fitness function, *W*, should be a function of *B* and *V*, satisfying the following properties: *W*(*B*,*V*) is a nonnegative function, such that *W*(*B*,*V*) = 0 if and only if *B* = 0 or *V* = 0. Moreover, the fitness function should be an increasing function on *B* and *V* for all nonnegative values of variables [14], [23]. We suggest using the following fitness function, satisfying the selected properties:

$$W=B^{\alpha }V^{\beta },\;\text{with}\;\alpha >0\;\text{and}\;\beta >0.$$ (3)

First of all, note that the form (3) of the fitness function reflects the fact that both fitness components are essential for the colony's evolution and survival [5]. Furthermore, this form of fitness implies the following essential property [14]. Assume that the colony consists of two cells only: one cell has a high level of viability and a low level of fecundity, while the other cell has a low level of viability and a high level of fecundity. Each cell by itself has a low fitness, but together within the colony, the cells can interact with each other, hence, achieve a higher level of fitness. Moreover, the form (3) of the fitness function is a generalization of the fitness functions that have been considered in a number of recent works [14], [15], [26], and [27]. For example, with *α* = *β* = 1, we get the fitness function that has been considered by Michod et al. [14]. If the parameters *α* and *β* are interpreted as inverses of the time for growth, we get the fitness function proposed by Maliet et al. [27], where the time for growth represents the time that the cell is growing during its life cycle. If the parameter *α* is interpreted as an inverse of the generation time, we get the fitness function introduced by Solari et al. [15]. Another interpretation of these parameters stems from Herron et al. [26]. These parameters reflect the ‘importance’ of the fecundity and viability contributions correspondingly to the fitness of the colony. They can be also viewed as the rates of fecundity and viability, respectively. Herron et al. [26] have considered the population of Volvocalean green algae in mixed and still environments. Still environment can be seen as an environment in which viability (i.e., motility) is supposed to be more important than in a mixed environment. This means that the value of the exponent *β* in still environment should be higher than that in mixed environment.

We will now introduce the parameter *C* as the amount of resources available to the colony. It would be reasonable to assume that in some cases the amount of available resources is greater than the amount of resources sufficient for the colony’s well-being (i.e., the environment does not restrict the colony’s well-being and does not influence its optimal reproductive strategy or motility). Thus, we can represent the relationship between fecundity, viability and environmental factors using the following inequality:

f(B,V)≤C. (4)

For the sake of simplicity, we assume that the function *f* has the following linear form:

$$k_{1}B+k_{2}V\leq C.$$ (5)

Inequality (5), as well as its parameters, has simple biological interpretations. Since *C* represents the amount of resources available to the colony and there are only two components of fitness, fecundity and viability, it is natural to assume that the colony divides the resource *C* between these two components. The parameter *k*_1_ represents the amount of resources necessary to produce one unit of fecundity and *k*_2_ represents the amount of resources necessary to produce one unit of viability. Obviously, *k*_1_ > 0, *k*_2_ > 0 and *C* > 0.

We can now formulate mathematically the fitness optimization problem. Consider a colony of identical cells. Each cell can contribute to fecundity and viability. For each cell there is an intrinsic relationship (trade-off) that links fecundity and viability. For all cells this relationship is the same due to the fact that the cells are identical. Further in this section, we assume that the trade-off constraints (1) are binding for all cells of the colony. The case of binding trade-off constraints is accounted for by changing inequalities by equalities (i.e., *v*_*i*_ = *φ*(*b*_*i*_)) in (1). In the following sections we will show how the solution of our problem changes if the assumption of binding trade-offs is ruled out. The fitness of the colony can be described by Eq (3). This equation takes into account the ‘importance’ of each component of the fitness measurement. Also, there is a composite resource consumed by a colony that affects the colony’s well-being according to Inequality (5). The colony’s optimal strategy, (b*,v*), consists of maximizing the fitness of the colony subject to all restrictions and conditions mentioned above (note that we can search for b* only because v* can be calculated directly from b* using the trade-off function). Thus, we obtain the following mathematical programming problem:

$$\{\begin{array}{c} W={\left(\sum_{i=1}^{N}b_{i}\right)}^{\alpha }{\left(\sum_{i=1}^{N}v_{i}\right)}^{\beta }\to \max_{b,v}\\ v_{i}=\varphi (b_{i}),i=1,\ldots ,N,\\ k_{1}\sum_{i=1}^{N}b_{i}+k_{2}\sum_{i=1}^{N}v_{i}\leq C,\\ b_{i}\geq 0,v_{i}\geq 0,i=1,\ldots ,N. \end{array}.$$ (6)

Our goal here is to find the set of all optimal fitness strategies for a given colony and make conclusions about its specialization. According to [14], the cell *i* specializes in soma if and only if *v*_*i*_ *= v*^*max*^, and the cell *i* specializes in germ if and only if *b*_*i*_ *= b*^*max*^.

The curvature of trade-off functions is one of the key parameters of functional specialization. According to Michod et al. [14], this curvature is concave in small-sized colonies and becomes gradually convex as the size of the colony increases. Initial cost of reproduction influences the curvature of the trade-off function, making it more ‘convex-like’ [14]. In the next sub-section we provide general statements characterizing our model, which hold for colonies of any size. Further we describe how these statements work with different types of trade-off functions: convex (for large-sized colonies), linear and concave (for small-sized colonies).

#### General statements

In this and the next sections we assume that the domain of Problem (6) is nonempty, i.e., we consider an environment, such that the amount of composite resources available in this environment is sufficient for the colony in order to live and reproduce. There are three possible cases when looking for the solution of Optimization Problem (6). Consider the problem of optimization of the fitness function with respect to given trade-off constraints. After adding to it the resource constraint, we get Problem (6). Two cases are possible here: (a) the addition of the resource constraint does not change the solution of the problem under study (we call it Case 1) and (b) this addition changes the solution of the problem under study. Let us describe in more detail the case (b). Assume that we have no any trade-off constraint, and the only constraint available is the resource constraint (5) (of course, we also assume that *B* > 0 and *V* > 0). In this case we can find the set *O* of all optimal strategies of the colony. After that, we add the trade-off constraints expressed as equalities, and thus get Problem (6). Obviously, some points of the set *O* might be unattainable due to the trade-off constraints. Let *A* be the set of points from *O* that remain attainable after adding these constraints. Two cases are possible here (we call them Cases 2 and 3 correspondingly). Case 2: the set *A* is nonempty. It means that the trade-off constraints influence the set of all optimal strategies (because they reduce *O* to *A*), but do not influence the optimal value of fitness. The only constraint that influences the optimal value of fitness is the resource constraint. Case 3: the set *A* is empty. It means that the trade-off constraints influence both the set of all optimal strategies and the optimal value of fitness. Here, both the trade-off and resource constraints have impact on the optimal value of fitness. Clearly, the addition of the trade-off constraints leads to a decrease in the optimal value of the fitness function. Further we provide a more formal description of Cases 1–3. The proofs of all our results are presented in Appendices A and B in S1 Appendix.

*Case 1*. This case implies that the resource constraint does not influence the colony’s well-being. This means that we have a resource restriction such that an optimal fitness strategy for the colony without any resource restriction continues to be available to the colony regardless of environmental constraints.

*Case 2*. Consider the following set of fecundity values:

$$A=\left\{b\in R^{N}|0\leq b_{i}\leq b^{\max}{}_{i},B(b)=\frac{C}{k_{1}\left(1+\frac{\beta }{\alpha }\right)}\;\text{and}\;V(b)=\frac{C}{k_{2}\left(1+\frac{\alpha }{\beta }\right)}\right\}.$$ (7)

The second case implies that the resource constraint influences the colony’s well-being and that the set *A* is not empty. Under this assumption, the set *A* is the solution of Optimization Problem (6). In Appendix B in S1 Appendix we prove that the elements of the set *A* (and only these elements) represent the solution of Problem (6). Moreover, we can calculate the maximum fitness W* that the colony can reach (i.e., the optimal fitness that the colony can reach by choosing any reproductive strategy from the set A):

$$W^{*}={\left(\frac{\alpha }{k_{1}}\right)}^{\alpha }{\left(\frac{\beta }{k_{2}}\right)}^{\beta }{\left(\frac{c}{\alpha +\beta }\right)}^{\alpha +\beta }.$$ (8)

We can conclude that the optimal viability and the optimal fecundity of the colony increase as the amount of available resources increases. The optimal fecundity of the colony decreases as the amount of resources necessary to produce one unit of fecundity, i.e., the variable *k*_1_, increases. The optimal viability of the colony decreases as the amount of resources necessary to produce one unit of viability, i.e., the variable *k*_2_, increases. The optimal fecundity of the colony increases and the optimal viability of the colony decreases as the relative ‘importance’ of fecundity to viability increases.

Thus, the best fitness the colony can get is an increasing function of the amount of available resources and a decreasing function of the amount of resources necessary to produce one unit of fecundity (or one unit of viability). The relationship between the optimal fitness and the parameters *α* and *β* is more complex. It is obtained by taking the following derivative:

$$\frac{\partial W^{*}}{\partial \alpha }=W^{*}\;\text{ln}\;\left[\frac{C}{k_{1}}\left(\frac{\alpha }{\alpha +\beta }\right)\right].$$ (9)

So, we can conclude that the optimal fitness is an increasing function of the ‘importance’ of fecundity if and only if

$$\frac{k_{1}}{C}\left(1+\frac{\beta }{\alpha }\right)<1.$$ (10)

In other words, the optimal fitness increases as the ‘importance’ of fecundity increases if and only if the relative ‘importance’ of fecundity is high and the amount of recourses necessary to produce one unit of fecundity, expressed in terms of all available resources, is low (i.e., it is not very expensive to produce a unit of fecundity and the ‘importance’ of fecundity is high).

Let us now consider the solutions of our problem, i.e., the set *A*. It has the following structure: the set *A* consists of a number of subsets; each of these subsets is a connected set; any two of these subsets have no intersection. The model is robust within each optimal subset, and the colony under study has more opportunities for adaptation because some of its cells can change slightly their optimal contributions to viability and fecundity without loss in fitness. In all optimal subsets, the levels of fecundity and viability of each cell are located in limited ranges, specific to each cell. This result reflects the fact that some cells in the colony may lose the potential ability to achieve, for example, a high level of fecundity, but they do not lose their capacity to perform a reproductive function. This case corresponds to an intermediate state between unspecialized colonies and full-specialized multicellular organisms.

*Case 3*. The third case implies that the resource constraint influences the colony’s well-being and the set *A* is empty. Here the mathematical solution becomes more complex and depends on the parameters of the model (for more details, see Appendix B in S1 Appendix). Further we show that this case provides the strongest incentives for the emergence of specialization in small-sized colonies and damages specialized structures in large-sized colonies.

In the next sections we will describe how our model can be applied in the context of different curvatures of trade-off functions. Moreover, we will present the set of all solutions of our mathematical programming problem using 3-D plots. In these plots we assume that we have three groups of cells within the colony such that all cells from each group contribute to the same levels of fecundity and to the same levels of viability (in other words, we have three ‘aggregate’ cells in the colony). This assumption is used to illustrate our general results graphically.

It is worth noting that our general results presented in this sub-section also hold for non-additive forms of the viability function. In Appendix B in S1 Appendix we provide a general proof of these results, which works for both additive and non-additive viability functions. Moreover, following Michod et al. [14], we can conclude that all theoretical results regarding the convex form of the viability function presented in this paper hold as well without the assumption of additivity (for more details see the sub-section Convex trade-off).

#### Convex trade-off

Assume that the trade-off function *φ*(*b*) is strictly convex on its domain.

*Case 1*. In this case an optimal strategy b* implies that all cells (or all cells but one) should be specialized. According to the model introduced by Michod et al. [14], with *α = β =* 1, a half of the cells should specialize in germ and a half in soma. Thus, our model can be viewed as a generalization of the model formulated in [14]. In our model, the number of cells specialized in germ (soma) depends on the ratio (*α/β*). For the colonies with a high relative importance of fecundity, the ratio (*α/β*) is high and, hence, reproductive cells are beneficial. For the colonies with a low relative importance of fecundity, the ratio (*α/β*) is low and, hence, somatic cells are beneficial. This theoretical observation is supported by the empirical fact that in still environment (i.e., when viability is more important than fecundity) the number of somatic cells is generally higher than in mixed environment [26].

**Fig 1:**
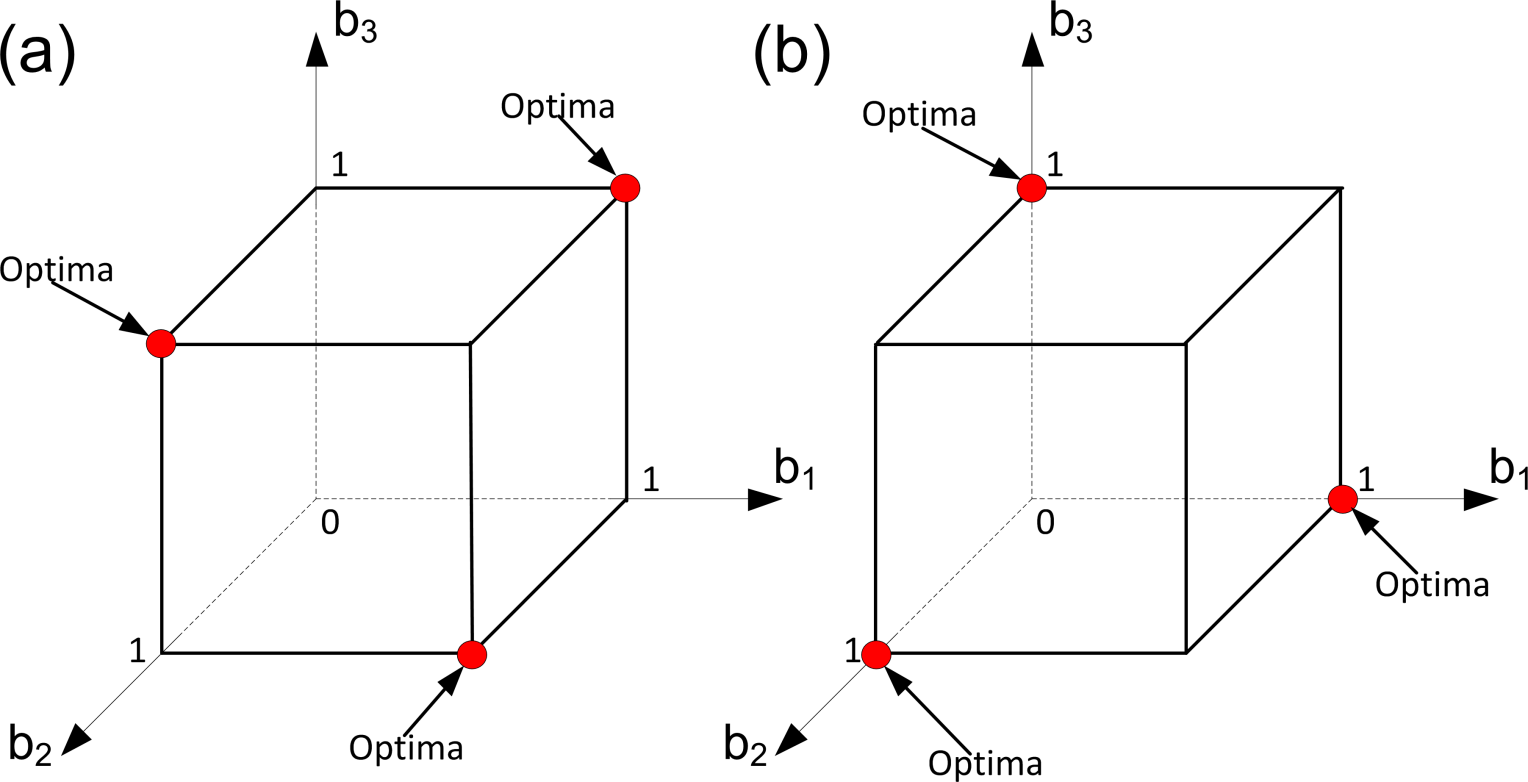
Evolution of germ and soma in colonies with identical cells: A convex trade-off, a resource constraint and different ‘importances’ of viability and fecundity (Case 1). Here we assume that we have three groups of cells within the colony such that all cells from each group have the same level of fecundity and the same level of viability (i.e., we have three ‘aggregate’ cells in the colony). Optimal strategies of the colony are colored in red. The level of specialization in soma and germ depends on the relative ‘importance’ of fecundity to viability. In both panels, *k*_1_ *= k*_2_ *=* 1, *α =* 1, *C =* 4 and *φ =* (*b-*1)^2^. In panel (a), *β =* 0.5. In panel (b), *β* = 2.

This case is illustrated in Fig 1. We will show how the solutions of the problem change in response to decreasing the ratio *α/β*. To do so, we assume that all other parameters of our model are fixed. In Fig 1 (panel a) fecundity is more important than viability, and in Fig 1 (panel b) viability is more important than fecundity. In each panel of Fig 1 the domain of the corresponding sub-problem of Problem (6) is plotted in the (*b*_1_, *b*_2,_ *b*_3_) space and the corresponding solution is depicted. Here each axis represents a reproductive effort of some ‘aggregate’ cells of the colony. Since we are in Case 1, the resource constraint does not influence the colony’s well-being. Consequently, only the trade-off constraints determine the domain of Problem (6). In the selected space these constraints form the cube [0, *b*^*max*^]^3^. We can see that in both panels of Fig 1 there exist three optimal strategies and within each of these strategies the colonies are full-specialized. The difference in the value of the (*α/β*) ratio leads to the difference in the number of ‘aggregate’ cells specialized in a specific fitness component. Thus, in Fig 1A with a large value of the ratio (*α/β*), each optimal strategy implies that two “aggregate” cells should specialize in germ, and one in soma. On the opposite, in Fig 1B with a small value of the ratio (*α/β*), each optimal strategy implies that only one ‘aggregate’ cell should specialize in germ, and two in soma.

**Fig 2:**
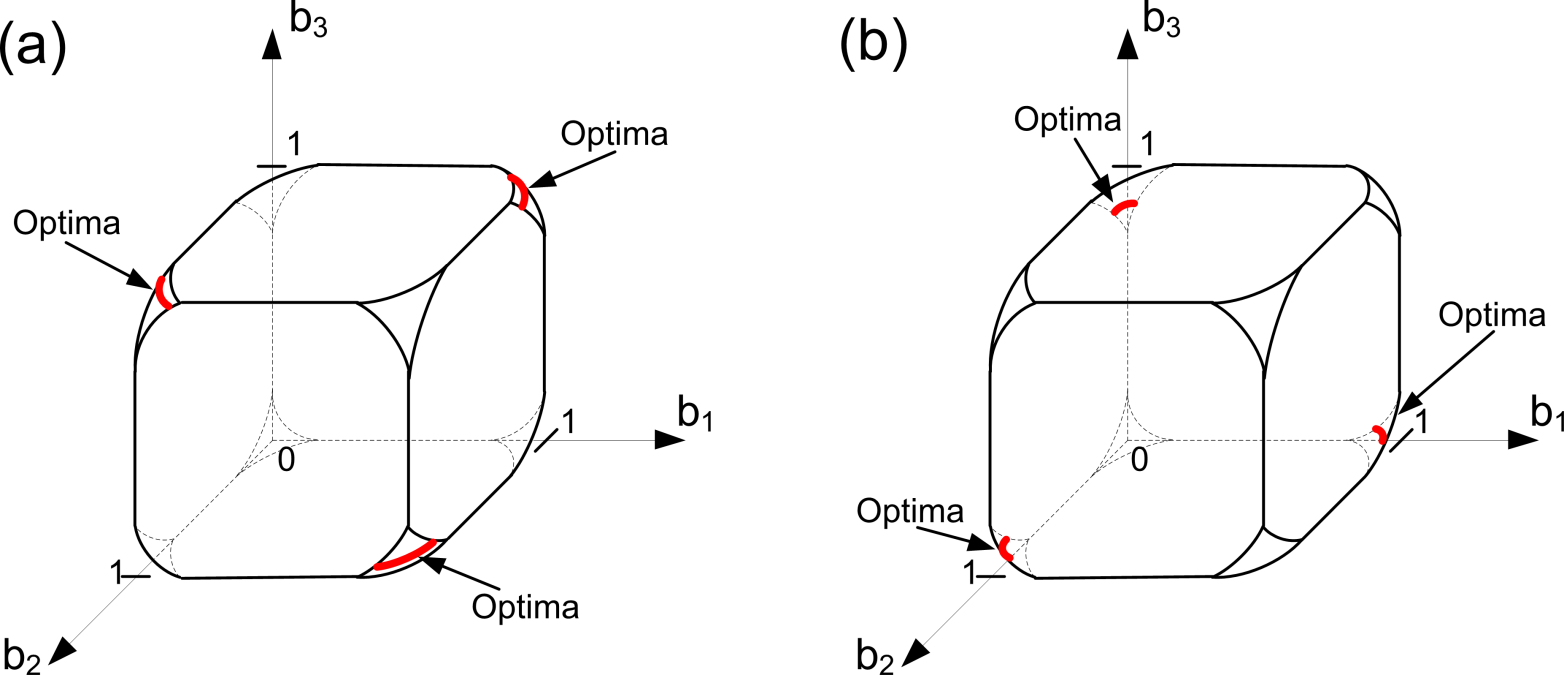
Evolution of germ and soma in colonies with identical cells: A convex trade-off, a resource constraint and different ‘importances’ of viability and fecundity (Case 2). Here we assume that we have three groups of cells within the colony such that all cells from each group have the same level of fecundity and the same level of viability (i.e., we have three ‘aggregate’ cells in the colony). Optimal strategies of the colony are colored in red. The level of specialization in soma and germ within the set *A* depends on the relative ‘importance’ of fecundity to viability. In both panels, *k*_1_ *= k*_2_ *=* 1, *α =* 1, *C =* 2.85 and *φ =* (*b-*1)^2^. In panel (a), *β =* 0.5. In panel (b), *β* = 2.

*Case 2*. Fig 2 illustrates this case. In this case the set *A* represents the solution of the problem under study. Clearly, the geometry of *A* depends on the value of the ratio (*α/β*). Since we are in Case 2, the resource constraint defines the domain of Problem (6) along with the trade-off constraints. Graphically, it means that the resource constraint truncates the trade-off cube so that the domain of Problem (6) is a truncated cube whose corners are cut out. The case presented in Fig 2 can be derived from the case presented in Fig 1 by reducing the amount of available resources. Thus, in Fig 2B viability is more important than in Fig 2A. In both cases, the set of optimal solutions (set *A*) consists of three disconnected subsets (they are colored in red). The difference between Fig 2A and 2B is that the optimal contribution of the colony to viability in Fig 2B is higher than in Fig 2A (i.e., in Fig 2B more cells are predisposed to the soma specialization than in Fig 2A). It is worth noting that at most one ‘aggregate’ cell can be specialized here.

**Fig 3:**
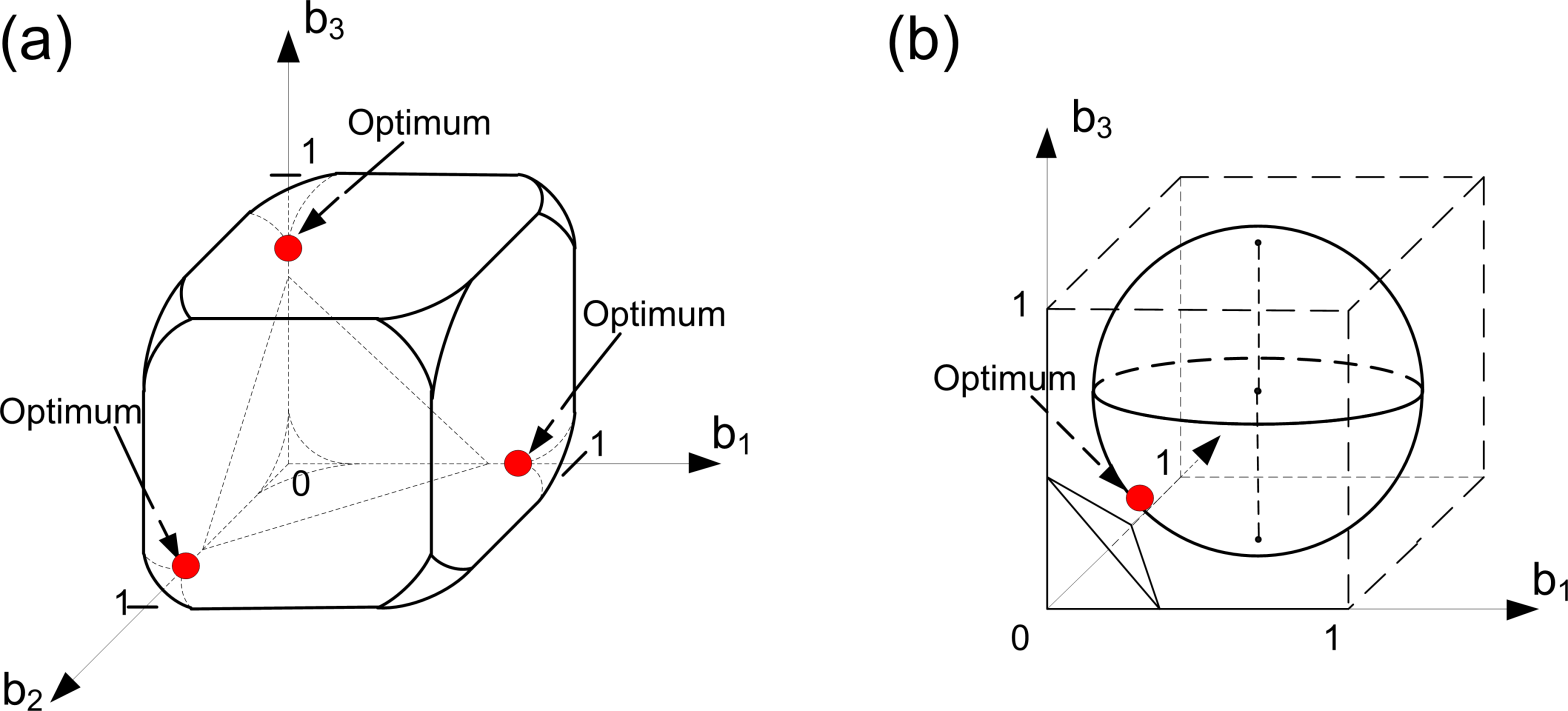
Evolution of germ and soma in colonies with identical cells: A convex trade-off, a resource constraint and different ‘importances’ of viability and fecundity (Case 3). Here we assume that we have three groups of cells within the colony such that all cells from each group have the same level of fecundity and the same level of viability (i.e., we have three ‘aggregate’ cells in the colony). Optimal strategies of the colony are colored in red. The evolution of structural complexity in unspecialized large-sized colonies can lead to the emergence of soma specialization (panel (a)) as well as to unspecialized optimal states in the case of environmental quality degradation (panel (b)). In both panels, *k*_1_ *= k*_2_ *=* 1, *α =* 1 and *φ =* (*b-*1)^2^. In panel (a), *β =* 4 and *C =* 2.85. In panel (b), *β* = 4 and *C =* 2.5.

*Case 3*. Fig 3 illustrates this case. We will show that Case 3 emerges either in a good environment and is then characterized by a non-zero level of specialization at the optimum or in a bad environment and is then characterized by an unspecialized optimal strategy. Since we are in Case 3, the domain of Problem (6) is still a truncated trade-off cube in the space (*b*_1_, *b*_2,_ *b*_3_).

First of all, consider the case presented in Fig 3A. It can be derived from the case presented in Fig 2B in the following way. Consider the case illustrated in Fig 2B. Suppose that the ‘importance’ of viability increases in a way that the set *A* becomes empty. In this case, the optimal strategies of the colony include the points from the set of all available strategies of the resource constraint surface, such that they are the closest to the following set: $\left\{b\in R^{N}|B(b)=\frac{C}{k_{1}\left(1+\frac{\beta }{\alpha }\right)}\right\}$(this set is depicted in Fig 3A; geometrically, it represents a simplex inside the truncated cube; this simplex has no intersections with the boundary of the truncated cube formed by the resource constraint). On one hand, this leads to a loss in fitness, but on the other hand, to an increase in specialization. We can see that there are three optimal strategies here. Each of them requires exactly two ‘aggregate’ cells to specialize in soma. Thus, the level of specialization of the colony increases. This case can occur during the evolution of Volvocalean green algae, when soma has to evolve first [15]. Hence, the functional specialization in Volvocalean green algae has the following form: GS (unspecialized colonies)–GS/S (specialization in soma only)–G/S (fully specialized colonies). Moreover, this case shows that the evidence that the process of transition goes through the path GS–GS/S can be explained not only in terms of enhancing the colony size [15]. Consider the following fitness function, originally studied in [15], which is a special case of the fitness function (3),

$$W=BV^{T}.$$ (11)

Let us now assume that initially we had a low generation time (*T*) and a resource constraint similar to that presented in Fig 2B. Furthermore, we suppose that the structural complexity of the colony evolves (without changes in its size). It requires colony to reproduce the structure that becomes more complex and, consequently, can lead to an increase in generation time. Therefore, we get the case illustrated in Fig 3A, when soma has to evolve first.

The case presented in Fig 3B can be derived from the case presented in Fig 3A in the following way. Let us consider the case illustrated in Fig 3A. We assume that the amount of available resources *C* decreases significantly so that the resource constraint becomes so strong that it is now the only factor that influences the domain of Problem (6). Geometrically, it means that the truncated cube formed by the intersection of the trade-off and resource constraints is transformed into a ball determined by the resource constraint only. In this case, the beneficial soma-specialized strategies become unreachable to the colony, which becomes unspecialized. This means that the changes in structural complexity in unspecialized large-sized colonies can lead to the emergence of soma specialization (Fig 3A) as well as to unspecialized states in the case of environmental quality degradation (Fig 3B).

#### Linear trade-off

Let us now assume that the trade-off function *φ*(*b*) is linear and has the following form:

$$\varphi (b)=v^{\max}-\gamma b,\;\text{where}\;\gamma >0.$$ (12)

A linear function is the simplest type of trade-off function which can be considered. Due to its simplicity, there are fewer effects that occur for this curvature of the trade-off function. For instance, Case 2 discussed in the previous sub-section does not take place here for most of the parameter values. Moreover, the resource constraint (5) can be represented under the following form

$$B\times sign(k_{1}-k_{2}\gamma )\leq \frac{C-k_{2}Nv_{\max}}{\left|k_{1}-k_{2}\gamma \right|}.$$ (13)

*Case 1*. The colony of cells behaves as a single cell as in [14]. The maximum fitness is obtained for all strategies b*, *0 ≤ b*_i_* *≤ b*^*max*^, such that:

$$B(b*)=\frac{Nb_{\max}}{1+\frac{\beta }{\alpha }}.$$ (14)

The maximum fitness is equal to

$$W*={\left(\frac{N}{\alpha +\beta }\right)}^{\alpha +\beta }{\left(\alpha b_{\max}\right)}^{\alpha }{\left(\beta v_{\max}\right)}^{\beta }.$$ (15)

The necessary and sufficient condition for the emergence of this case is the following

$$C\geq \frac{\alpha }{\alpha +\beta }k_{1}Nb_{\max}+\frac{\beta }{\alpha +\beta }k_{2}Nv_{\max}$$ (16)

This inequality suggests that the resource constraint does not influence the colony’s well-being if and only if the amount of available resources is sufficient to produce some fractions of the maximal possible values of the colony’s fecundity and viability. These fractions are proportional to the ‘importance’ of fecundity and viability, respectively.

**Fig 4:**
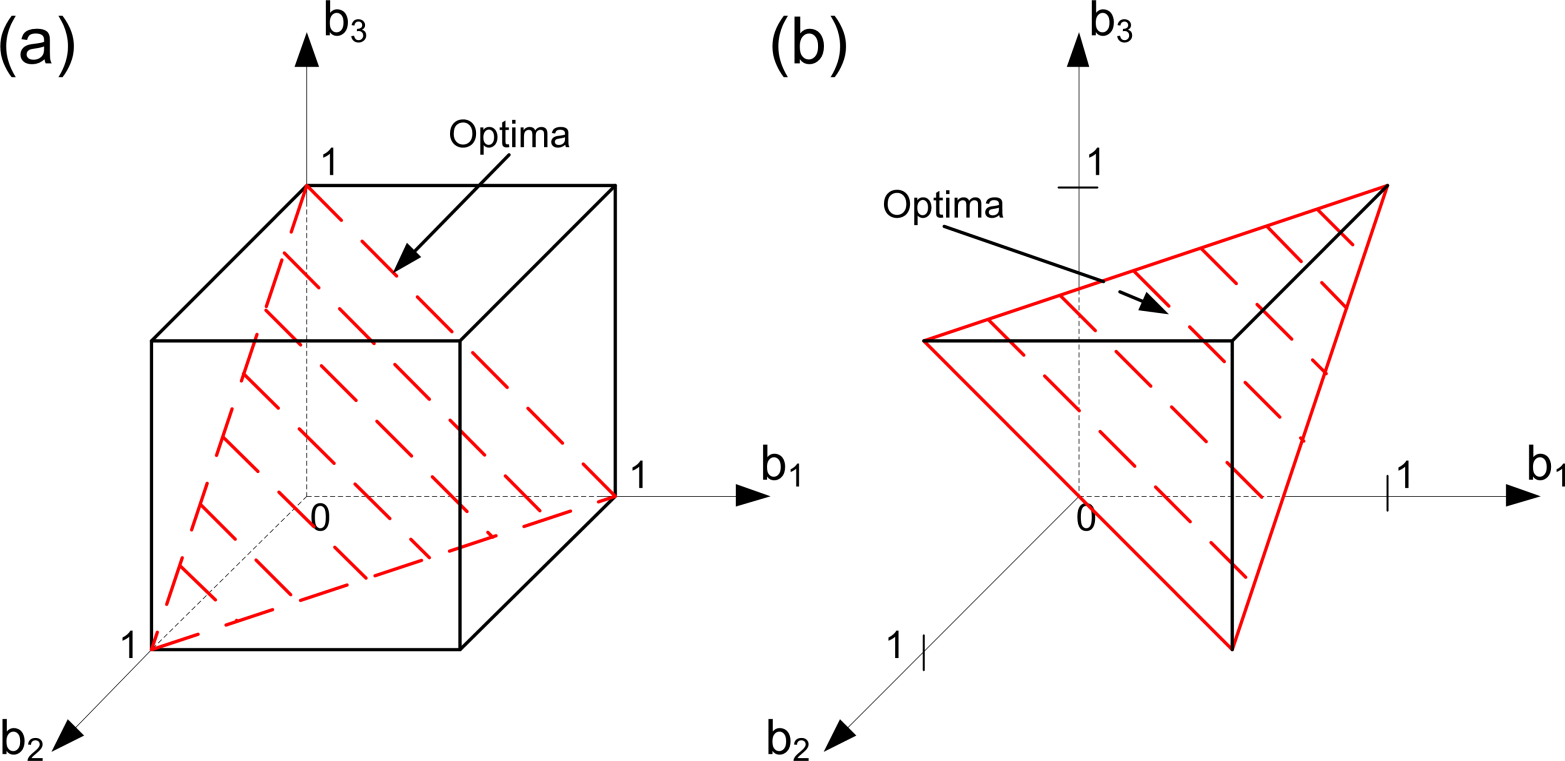
Evolution of germ and soma in colonies with identical cells: A linear trade-off, a resource constraint and different ‘importances’ of viability and fecundity. Here we assume that we have three groups of cells within the colony such that all cells from each group have the same level of fecundity and the same level of viability (i.e., we have three ‘aggregate’ cells in the colony). In panel (a), Case 1 is illustrated. In panel (b), Case 3 is illustrated. With a linear trade-off there is no Case 2 in the model (i.e., it means that the set of parameters that lead to Case 2 in the space of all possible parameters is a set of measure zero). Optimal strategies of the colony are colored in red. With a linear trade-off, identical colony behaves as a single cell. In all panels, *α* = 1, *β* = 2, *k*_1_ = 1, *C* = 4 and *φ =* 1-*b*. In panel (a), *k*_2_ *=* 1. In panel (b), *k*_2_ *=* 2.

This situation is illustrated in Fig 4A. Since we consider Case 1, the domain of Problem (6) is the trade-off cube. The solutions of this problem can be found using Eq (14). Geometrically, the solutions are located on the intersection of the trade-off cube and a plane determined using Eq (14). This intersection forms a simplex inside the cube. Each strategy from this simplex will be optimal. We can see that in this case the specialized states are optimal as well as unspecialized ones.

*Case 3*. The colony of cells behaves as if it was a single cell too. The maximum fitness is obtained for all strategies b*, *0 ≤ b*_i_* *≤ b*^*max*^, such that:

$$B(b*)=\frac{C-k_{2}Nv_{\max}}{k_{1}-k_{2}\gamma }.$$ (17)

The maximum fitness here is as follows:

$$W*={\left(\frac{C-k_{2}Nv_{\max}}{k_{1}-k_{2}\gamma }\right)}^{\alpha }{\left(\frac{k_{1}Nv_{\max}-\gamma C}{k_{1}-k_{2}\gamma }\right)}^{\beta }.$$ (18)

The necessary and sufficient condition for the emergence of this case is the following:

$$\min\left\{k_{1}Nb_{\max},k_{2}Nv_{\max}\right\}\leq C<\frac{\alpha }{\alpha +\beta }k_{1}Nb_{\max}+\frac{\beta }{\alpha +\beta }k_{2}Nv_{\max}.$$ (19)

It is worth noting that if the left-hand side of this inequality does not hold, the colony has no possibility to survive. Also, if *k*_1_*b*_*max*_ = *k*_2_*v*_*max*_, Inequality (19) does not hold hence Case 3 does not occur here.

Case 3 is illustrated in Fig 4B. In the linear case the resource constraint is represented by a plane (see Eq 13). This plain cuts out a part of the trade-off cube thereby forming the domain of Problem (6). Moreover, all the states from the intersection of the trade-off cube, and the resource plane are optimal. As in Case 1, the colony can be unspecialized in some optimal states and some level of specialization can occur in the other optimal states. Thus, we can conclude that the solution structures of a linear trade-off model do not alter with the changes in the model's parameters and that there is no major difference between Cases 1–3 here.

#### Concave trade-off

Let us assume that the trade-off function *φ*(*b*) is strictly concave on its domain.

*Case 1*. Here we consider the strategy that implies that each cell in the colony has the same level of fecundity (i.e., the same level of viability) b*, such that:

$$\frac{d\varphi }{db}(b*)=-\frac{\alpha }{\beta }\frac{V(b*)}{B(b*)}.$$ (20)

It is easy to show that there is a single point b* satisfying Eq (20), such that 0 < b* *< b*^*max*^. Moreover, this point represents the single optimal strategy of the colony at hand. Eq (20) can be rewritten in the following way:

$$\varepsilon_{VB}(b*)=\frac{dV(b*)}{dB(b*)}\times \frac{B(b*)}{V(b*)}=-\frac{\alpha }{\beta }.$$ (21)

The parameter *ε*_*VB*_ is the elasticity of the group’s viability relative to fecundity. Thus, the optimal strategy for the colony requires the elasticity of the group to be equal to the relative ‘importance’ of viability to fecundity. This means that at the optimum, in order to increase the group’s viability by one percent, it is necessary to decrease the group’s fecundity by the relative ‘importance’ of viability to fecundity percent.

**Fig 5:**
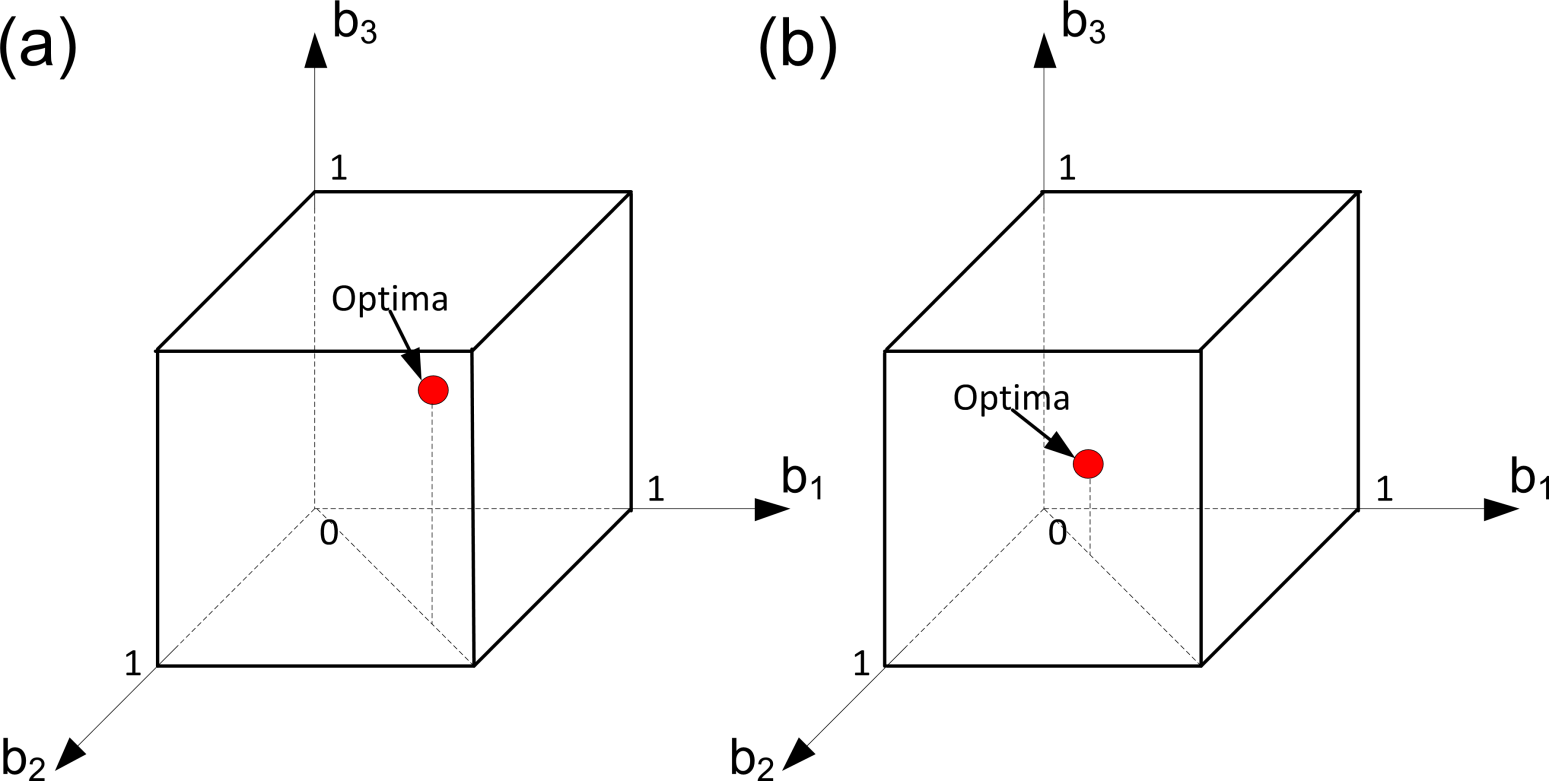
Evolution of germ and soma in colonies with identical cells: A concave trade-off, a resource constraint and different ‘importances’ of viability and fecundity (Case 1). Here we assume that we have three groups of cells within the colony such that all cells from each group have the same level of fecundity and the same level of viability (i.e., we have three ‘aggregate’ cells in the colony). Optimal strategies of the colony are colored in red. In both panels, the colonies have a unique optimal strategy and the specialization does not occur. The contribution of the colony to the fitness components depends on the relative importance of fecundity to viability. In both panels, *α* = 1, *φ =* 1-*b*^2^, *C* = 8, *k*_1_ *=* 2 and *k*_2_ *=* 1. In panel (a), *β* = 0.5. In panel (b), *β* = 2.

Let us now consider Fig 5. The two panels of this figure show how the model's solution vary with respect to the changes in the (*α/β*) ratio. Since we are in Case 1, the domain of Problem 6 is a trade-off cube. According to Eq (20), the problem's solution should be located on the main diagonal of the cube. In panel (a) of Fig 5 fecundity is more important than in panel (b). Thus, Fig 5A illustrates a case where, at the optimum, cells invest more in fecundity than in Fig 5B.

**Fig 6:**
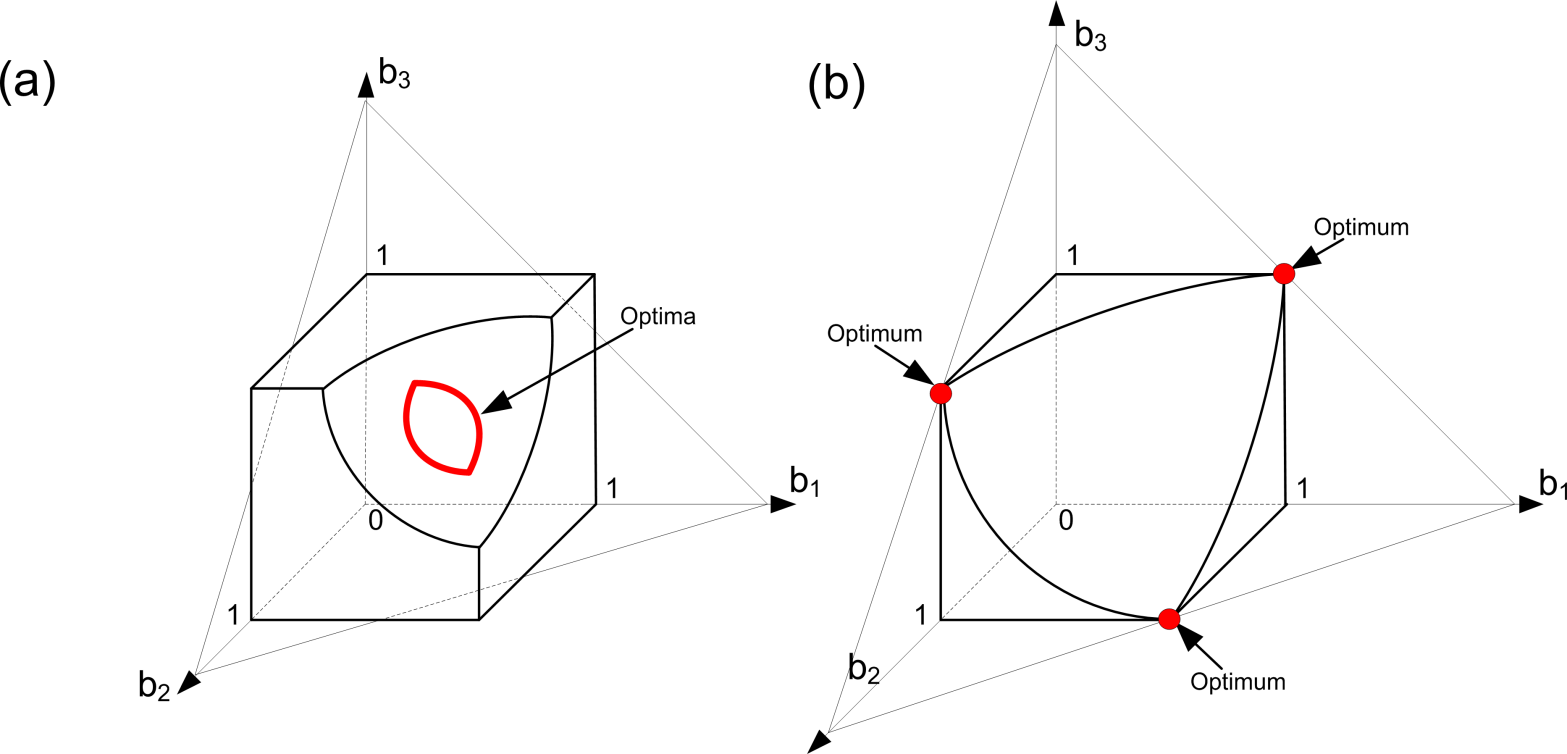
Evolution of germ and soma in colonies with identical cells: A concave trade-off, a resource constraint and different ‘importances’ of viability and fecundity (Case 2). Here we assume that we have three groups of cells within the colony such that all cells from each group have the same level of fecundity and the same level of viability (i.e., we have three ‘aggregate’ cells in the colony). Optimal strategies of the colony are colored in red. In panel (a) the optimal solutions represent a connected set (set *A*) of unspecialized states that provides opportunities for adaptation for the colony at hand. In panel (b), the set *A* is a three-point set. The trade-off between the production difficulties and the fitness benefits of one fitness component (panel (b)) is one of the main reasons for the emergence of specialization in small-sized colonies. In both panels, *α* = 1, *φ =* 1-*b*^2^, *k*_1_ = 2 and *k*_2_ = 1. In panel (a), *β* = 0.5 and *C* = 5.5, In panel (b), *β* = 0.25 and *C =* 5.

*Case 2*. The set *A* represents the solution of the problem under study. Fig 6 shows how the solution of the problem varies in response to the changes in the parameters. Consider panel (a) first. The resource constraint truncates the trade-off cube, thereby forming the domain of Problem (6). In this case viability is less ‘important’ than fecundity. Also, more resources for producing one unit of fecundity than for producing one unit of viability are required (due to this fact the truncated cube is asymmetrical and only one of its corners is cut out). We can see that the optimal solutions here form a connected set of unspecialized states (set *A*). Graphically, this set is located on the intersection of a part of the domain boundary defined by the resource constraint and the plane that provides the optimum of the fecundity function. The set *A* embodies the opportunities for adaptation for the colony at hand. If the relative importance of fecundity to viability grows (when the amount of available resources decreases slightly), we have the case shown in Fig 6B, where the specialization occurs. This is an extreme sub-case of Case 2, where the set *A* corresponds to a three-point set. The case presented in Fig 6B can be characterized by the two following properties. On one hand, the fecundity production requires more resources than the production of viability. On the other hand, fecundity yields significantly more benefits than viability. The emergence of specialization here is a consequence of the trade-off between the production difficulties and the fitness benefits of fecundity.

**Fig 7:**
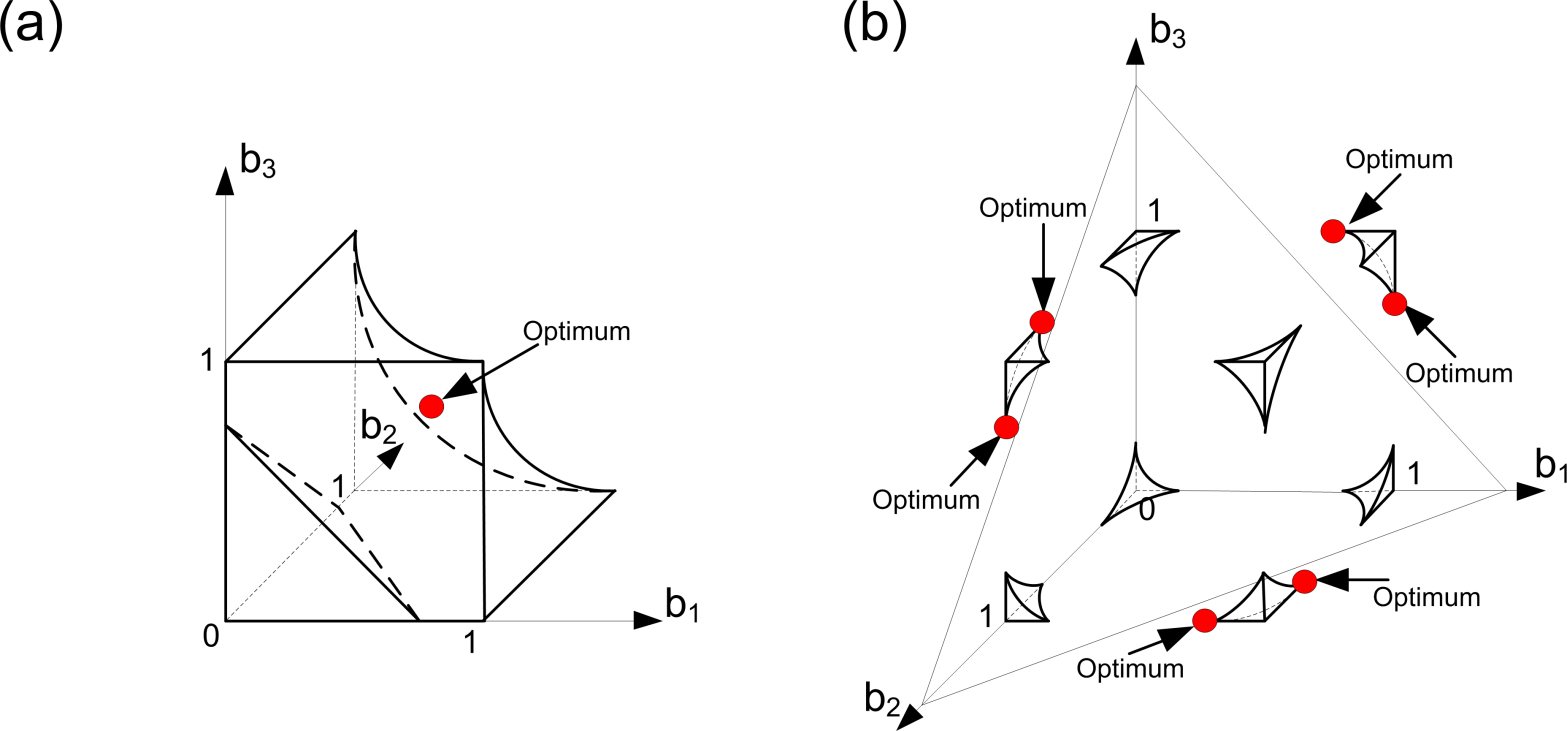
Evolution of germ and soma in colonies with identical cells: A concave trade-off, a resource constraint and different ‘importances’ of viability and fecundity (Case 3). Here we assume that we have three groups of cells within the colony such that all cells from each group have the same level of fecundity and the same level of viability (i.e., we have three ‘aggregate’ cells in the colony). Optimal strategies of the colony are colored in red. Unspecialized states (panel (a)) can be optimal as well as strategies with some level of specialization (panel (b)). Significant resource constraint (panel (b)) is one of the main reasons for the emergence of specialization in small-sized colonies. In both panels, *α* = 1, *φ =* 1-*b*^2^ and *k*_2_ *=* 1. In panel (a), *β* = 2, *C* = 5 and *k*_1_ *=* 2. In panel (b), *β* = 1, *C* = 3.12 and *k*_1_ *=* 1.

*Case 3*. We describe a general principle of finding solutions here: optimal strategies are the available strategies from the resource constraint surface such they yield the same level of total fecundity, and this level is the closest one to the total fecundity plane surface for the set *A*: $\left\{b\in R^{N}|B(b)=\frac{C}{k_{1}\left(1+\frac{\beta }{\alpha }\right)}\right\}$, among all available strategies belonging to the resource constraint surface defined by the equation *k*_1_*B*+*k*_2_*V* = *C*. Fig 7 illustrates this case and shows that unspecialized states can be optimal as well as strategies with some level of specialization. Here the domain of Problem (6) is an intersection of the trade-off cube and the resource constraint surface. Also, in Fig 7 we show the total fecundity plane of the set *A* in order to illustrate the general principle of finding optimal solutions in this case. The case presented in Fig 7A can be derived from the case presented in Fig 6A in the following way. Let us consider the case illustrated in Fig 6A. We suppose that the ‘importance’ of viability increases in a way that viability becomes more important than fecundity and the amount of available resources decreases slightly, as in the case illustrated in Fig 6B. Because the fecundity production requires more resources than the production of viability, and viability yields more benefits than fecundity, there is no trade-off between the production complexity and the fitness benefits of fecundity or viability. It implies that the colony has no incentive to reorganize the optimal structure of its contributions to the fitness components (i.e., the colony remains unspecialized). In Fig 7B another way of emergence of specialization is illustrated. Here we have the case where *k*_1_ *= k*_2_ *=* 1 and *α = β =* 1. This means that fecundity and viability have the same contribution to fitness and require the same amount of resources for production. Also, here we have a very low level of resources available (geometrically, it means that the resource constraint is so strong that only the eight corners of the trade-off cube constitute the problem domain). In this case the specialization occurs in response to a bad environmental quality. In order to survive and adapt, cells within the colony have to redistribute their efforts in a way that some of them become specialized.

### Optimization model for the colony of cells of different types

#### The model

In this section we will show how Optimization Problem (6) can be further generalized. In Problem (6) only identical cells are considered. Here we assume that some cells of the colony are not equivalent with respect to a given function (viability or fecundity) due to mutations, a specific developmental program or positional effects. This non-equivalence of cells can be expressed via the difference in the trade-off functions among cells of the same colony:

$$v_{i}=\varphi_{i}(b_{i}),\;\text{for}\;i=1,\ldots ,N.$$ (22)

Thus, each cell of the colony will have its own trade-off function, such that $b_{i}^{\max}\in R^{+}$, $b_{i}^{\max}<\infty$ and $\varphi_{i}:\left[0,b_{i}{}^{\max}\right]\to R^{+}\cup \left\{0\right\}$. As previously, we assume that all functions *φ*_i_ are continuous on $\left[0,b_{i}^{\max}\right]$, twice continuously-differentiable on $\left(0,b_{i}^{\max}\right)$, $\frac{d\varphi_{i}}{db_{i}}<0$ for all $b_{i}\in (0,b_{i}^{\max})$, and $\varphi_{i}(b_{i}^{\max})=0$ $\varphi_{i}(0)=v_{i}^{\max}$, $v_{i}^{\max}\in R^{+}$ and $v_{i}^{\max}<\infty$. We say that two cells have different types if and only if they have different trade-off functions. Thus, we can formulate the fitness optimization problem for the colony of cells of different types by replacing the trade-off functions of form (1) by those of form (22).

By solving Problem (6) with different types of trade-offs, we determine how positional effects, the ‘importance’ of tasks and the resource constraint influence the optimal strategy of the colony and its incentives to specialization. It is worth noting that the results provided in the sub-section General statements of the previous section are generic and hold for the optimization model with different types of cells too (see Appendix B in S1 Appendix for more details). Below, we describe how these general statements can be illustrated under different curvatures of trade-off functions which are due to the presence of positional effects. More precisely, we will discuss Case 1 only for each curvature, because all other cases (except Case 2 for linear trade-offs) preserve all qualitative results and properties of the model presented in the previous section. In other words, there are some factors that favor the evolution of division of labor. These factors emerge due to the resource constraint and different ‘importances’ of viability and fecundity (we described them in the previous section). Here we show only the specific effects of different trade-off functions (but still with different ‘importances’ of viability and fecundity). Cases 2 and 3 of the current model combine the properties of Cases 2 and 3 of the model presented in the previous section and those of Case 1 described in the next sub-section.

#### Convex trade-offs

Let us assume that all trade-off functions are strictly convex on their domains.

*Case 1*. In this case an optimal strategy b* implies that all cells, or all cells except one, should be specialized and that the number of cells specialized in germ (or soma) depends on cells positional effect and on the ratio (*α/β*). To show it, we follow the elegant and simple arguments provided in [14]. Consider an arbitrary strategy *b* = (*b*_1_,…,*b*_*i*_,…,*b*_*j*_,…,*b*_*N*_), where two cells, *i* and *j*, have intermediate fecundities, $b_{i}\in (0,b_{i}^{\max})$ and $b_{j}\in (0,b_{j}^{\max})$. For any positive real number *ε*, such that $0<\varepsilon <\min\{b_{i},b_{j},b_{i}^{\max}-b_{i},b_{j}^{\max}-b_{j}\}$, we consider two new strategies *b*^1^ = (*b*_*1*_,…,*b*_*i*_–*ε*,…,*b*_*j*_+ε,…*b*_*N*_) and *b*^2^ = (*b*_*1*_,…,*b*_*i*_*+ε*,…,*b*_*j*_–*ε*,…*b*_*N*_). We can see that *B*^*α*^(*b*) = *B*^*α*^(*b*^*1*^) = *B*^*α*^(*b*^*2*^), and due to the strict convexity of *V*, we can conclude that 2*V*(*b*)<*V*(*b*^*1*^)+*V*(*b*^*2*^*)*. Thus, either *V*^*β*^(*b*)<*V*^*β*^(*b*^2^) or *V*^*β*^(*b*)<*V*^*β*^(*b*^*2*^). It means that either *W*(*b*)*<W*(*b*^1^) or *W*(*b*)*<W*(*b*^2^). Therefore, we showed that the strategy *b* is not optimal. Importantly, we did not specify the form of *V* in this proof, but required only the strict convexity of all trade-off functions. It means that this result holds for non-additive viability functions as well.

**Fig 8:**
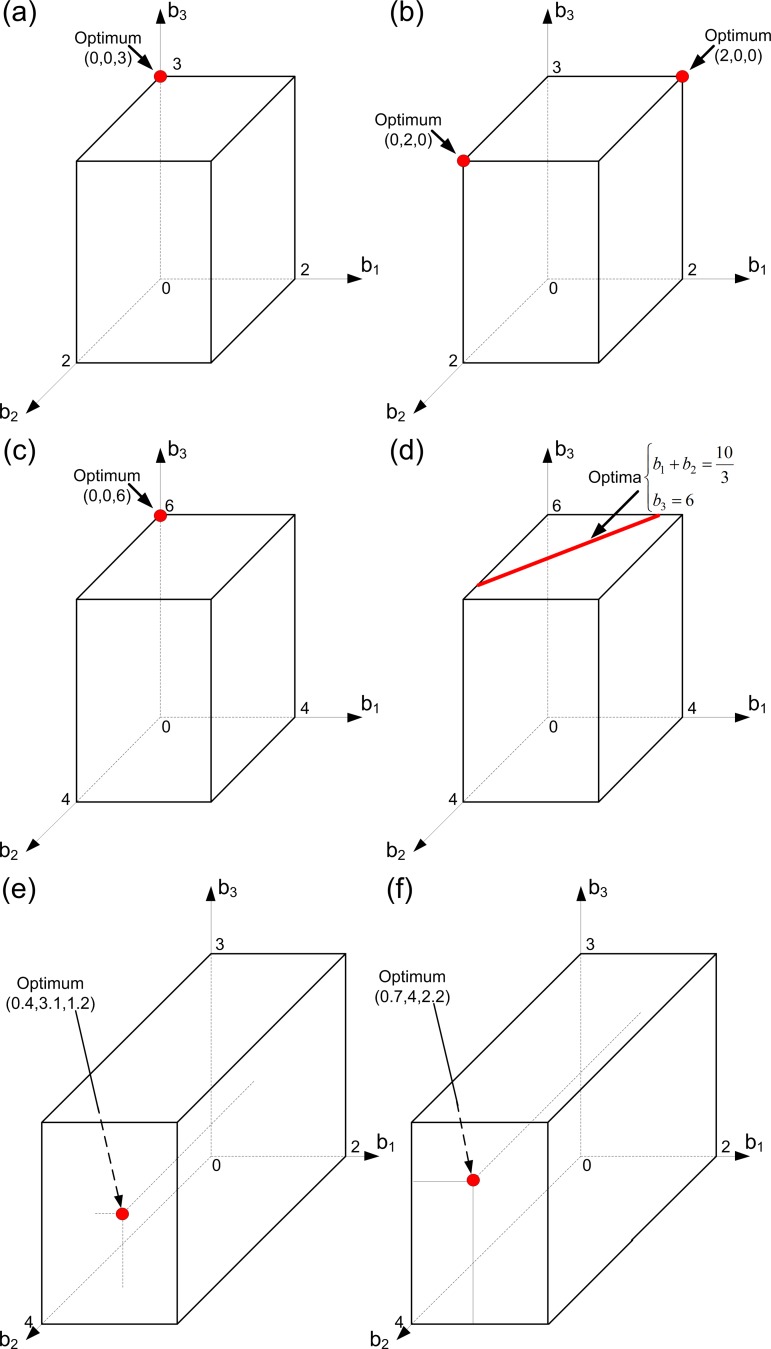
Evolution of germ and soma in colonies with: Positional effects, different curvatures of trade-off functions and different ‘importances’ of viability and fecundity (Case 1). Here we assume that we have three groups of cells within the colony such that all cells from each group have the same level of fecundity and the same level of viability (i.e., we have three ‘aggregate’ cells in the colony). The following 3-D panels are shown. In all panels, *α* = 1. In panels (a), (c) and (e), *β* = 2. In panels (b), (d) and (f), *β* = 0.5. In panels (a) and (b), *φ*_1_ *=* (*b*_1_-2)^2^, *φ*_2_ *=* (*b*_2_-2)^2^ and *φ*_3_ *=* (*b*_3_/√3 - √3)^2^. In panels (c) and (d), *φ*_1_ *=* 4-*b*_1_, *φ*_2_ *=* 4-*b*_2_ and *φ*_3_ = 2-*b*_3_/3. In panels (e) and (f), *φ*_1_ *=* 4-*b*_1_^2^, *φ*_2_ *=* 2-*b*_2_^2^/8 and *φ*_3_ *=* 3-*b*_3_^2^/3. Optimal strategies of the colony are colored in red.

This case is illustrated in Fig 8A and 8B. In Fig 8A, viability is more ‘important’ than fecundity and the third ‘aggregate’ cell is more predisposed to fecundity-performing. Therefore, an optimal strategy for the first and the second “aggregate” cells consists in the soma specialization, while the third cell should be specialized in germ. If the ‘importance’ of viability decreases so that fecundity becomes more “important” than viability, then we get the case illustrated in Fig 8B. Here the third cell remains specialized in germ due to positional effects. The increase in the ‘importance’ of viability forces the first, or the second, “aggregate” cell to switch its specialization from viability to fecundity.

#### Linear trade-offs

Let us assume that all trade-off functions are linear. Thus, they have the following form:

$$\varphi_{i}(b_{i})=v_{i}{}^{\max}-\gamma_{i}b_{i},\;\text{Where}\;\gamma_{i}>0,i=1,\ldots ,N.$$ (23)

*Case 1*. We assume that all parameters *γ*_*i*_ (*i =* 1,…,*N*) are different. We call this property “the assumption of strong differentiation of types”. In this case an optimal strategy b* implies that all cells, or all cells except one cell, are specialized. Moreover, this optimal strategy is unique, and the cells that specialize in germ are those with the lowest *γ*_*i*_, while the cells that specialize in soma are those with the highest *γ*_*i*_. To show this, we follow the approach provided in [14]. First of all, we can reformulate Problem (6) with different types of trade-offs, by replacing the original objective function: *W = B*^*α*^*V*^*β*^, by the following function: $W'=B^{\frac{\alpha }{\beta }}V$. Now we can consider an equivalent mathematical programming problem, in which for all feasible fecundity strategies *b* and *b*^1^, we have *W*(*b*^*1*^)–*W*(*b*)>0 if and only if *W'*(*b*^*1*^)–*W'*(*b*)>0. Let us consider an arbitrary strategy *b* = (*b*_*1*_,…,*b*_*i*_,…,*b*_*j*_,…,*b*_*N*_), where two cells, *i* and *j*, such that *γ*_*i*_ < *γ*_*j*_, have fecundities, $b_{i}\in [0,b_{i}^{\max})$ and $b_{j}\in (0,b_{j}^{\max}]$, respectively. Let us now select a real number *ε*, such that $0<\varepsilon <\min\{b_{j},b_{i}^{\max}-b_{i}\}$. If cell *i* increases its fecundity, while cell *j* decreases its fecundity, by the same amount *ε*, then the total fecundity of the colony does not change. However, the viability of the colony increases, and as a result, its fitness increases as well. More formally, we consider a new strategy *b*^*1*^ = (*b*_*1*_,…,*b*_*i*_*+ε*,…,*b*_*j*_*–ε*,…,*b*_*N*_). Because $B^{\frac{\alpha }{\beta }}(b)=B^{\frac{\alpha }{\beta }}(b^{1})$, we obtain: $W'(b^{1})-W'(b)=B^{\frac{\alpha }{\beta }}(b)\times (\gamma_{j}-\gamma_{i})\times \varepsilon >0$. It means that *W*(*b*^1^*)–W*(*b)>*0. Consequently, the strategy *b* is not optimal. Using similar arguments, we can prove that the solution of this problem is unique.

Furthermore, let us assume that there are cells *i*_1_,…,*i*_*k*_ in the colony, such that $\gamma_{i_{1}}=\ldots =\gamma_{i_{k}}=\gamma$. We can reduce this problem to the problem with “the assumption of strong differentiation of types”, by combining all cells *i*_1_,…,*i*_*k*_ into a single composite cell with fecundity $B=\sum_{l=1}^{k}b_{i_{l}}$ and viability $V=(\sum_{l=1}^{k}v_{i_{l}}^{\max})-\gamma \times B$.

This case is illustrated in Fig 8C and 8D). In Fig 8C, viability is more ‘important’ than fecundity and the third ‘aggregate’ cell is more predisposed to fecundity performing. Therefore, the optimal strategy for the first and the second “aggregate” cells is to be specialized in soma, and to be specialized in germ for the third cell. This means that positional effects here lead the colony to specialization. Without these effects in the linear case, the colony sees no difference between some unspecialized states and the states where some cells can be specialized (see the sub-section Linear trade-off in the previous section). If the ‘importance’ of viability decreases in a way that fecundity becomes more “important” than viability, we get the case illustrated in Fig 8D. Here we conclude that the third cell should remain specialized in germ due to positional effects. The first and the second cells behave as if they were one cell. This example suggests that there is a non-trivial interaction between positional effects and the ‘importance’ of the fitness components. If they act as one, as shown in Fig 8C, the specialization in the colony occurs. Otherwise, they compensate each other and the specialization occurs only partially, as shown in Fig 8D.

*Case 2*. In the sub-section Linear trade-off of the previous section we showed that in the linear case without positional effects, only Cases 1 and 3 can occur. However, if we introduce positional effects into the model with linear trade-offs, all three cases (Cases 1 to 3) become possible.

#### Concave trade-offs

Let us assume that all trade-off functions are strictly concave on their domains.

*Case 1*. We consider the strategy implying that each cell in the colony has the level of fecundity *b*_i_*, such that:

$$\frac{d\varphi_{i}}{db_{i}}(b_{i}{}^{*})=-\frac{\alpha }{\beta }\frac{V(b*)}{B(b*)},i=1,\ldots ,N.$$ (24)

If there exists a point b*, satisfying Eq (24), such that 0 < *b*_i_* < *b*_*i*_^*max*^ for all *i =* 1,…, *N*, then this point represents the unique optimal strategy of the colony under study, i.e., all cells in the colony remain unspecialized. However, the case when no points satisfy Eq (24), such that 0 < *b*_i_* < *b*_*i*_^*max*^ for all *i* = 1…*N*, can also occur. This case occurs when the relative ‘importance’ of viability to fecundity is high (low) enough or when the differentiation of types is strong. In these cases some cells of the colony should be specialized, whereas the other cells should have the level of fecundity *b*_i_* satisfying the condition similar to that of Eq (24) (see Appendix A in S1 Appendix). Note also, that high (low) relative ‘importance’ of viability to fecundity can pushes the colony to specialization only in the presence of positional effects, because without these effects (see the sub-section Concave trade-off in the previous section) the concave curvatures of trade-off functions do not lead to specialization in the colony. This case is shown in Fig 8E, where there is no specialization at the optimum. Then, we can also assume that the ‘importance’ of viability decreases in a way that viability becomes less “important” than fecundity (see Fig 8F). The optimal strategy for the second cell in this figure is to specialize in germ. On one hand, the second cell is predisposed to specialization in germ due to positional effects. On the other hand, this predisposition by itself has no impact on specialization in the colony (see Fig 8E). Fig 8F accounts for the case when the change in the relative ‘importance’ of viability to fecundity, combined with the presence of positional effects, causes the emergence of specialization. Consequently, we can say that positional effects influence the development of specialization in the colony directly (through predispositions of some cells to some tasks) or indirectly (through a high or low relative ‘importance’ of viability to fecundity supported by the presence of positional effects). In other words, positional effects and mutations in small-sized colonies can lead to the emergence of specialization without changes in size and/or in resource restrictions. Moreover, together with positional effects, the increase in the generation time or changes in the environment (from mixed to still) can lead to the emergence of soma specialization without changes in the size of the colony. Hence, we have shown that the GS–GS/S transition process during the evolution of Volvocalean green algae can be explained not only in terms of enhancing the colony size. Obviously, the size of the colony and the corresponding initial costs of reproduction are essential, but they are not the only factors causing the emergence of specialization.

### Generalization of the original model: Considering inequalities in trade-offs

Here we consider Problem 6 with different types of trade-offs, assuming that the trade-off relationships have the form (1), i.e., they are represented by inequalities. First of all, we need to point out that this problem has a nonempty domain, because the point (*b*,*v*) = (0,0) belongs to its domain for all possible values of parameters in the resource constraint. Further, we provide general results to describe the solutions of this problem and compare them to the results from the sub-section General statements of the previous section, where the trade-off relationships were represented by equalities. Here we also have three possible cases which are as follows:

*Case 1*'. Consider Problem (6) with different types of trade-offs represented by inequalities. First, we assume that there is no any resource constraint in the model, and identify the set of optimal strategies of the colony, {(b*, v*)}. These optimal strategies require the binding of all trade-off constraints. Let us now add the resource constraint to the model. Case 1' requires that at least one strategy from {(b*, v*)} would remain optimal. This case corresponds to the situation in which the resource constraint does not influence the colony's well-being. In other words, Case 1' occurs in environments of good quality.

*Case 2*'. This case implies that the resource constraint influences the colony’s well-being. Consider the following set of strategies:

$$A'=\left\{(b,v)\in R^{2N}|b_{i}\geq 0,0\leq v_{i}\leq \varphi_{i}(b_{i}),\sum_{i=1}^{N}b_{i}=\frac{C}{k_{1}\left(1+\frac{\beta }{\alpha }\right)}\;\text{and}\;\sum_{i=1}^{N}v_{i}=\frac{C}{k_{2}\left(1+\frac{\alpha }{\beta }\right)}\right\}.$$ (25)

Case 2' assumes that the set *A*' is not empty. This set includes the solutions of Problem (6) with different types of trade-offs represented by inequalities and the binding resource constraint. The set *A*' is similar to the set *A* described in the sub-section General statements of the previous section. In Appendix C in S1 Appendix we show that the elements of *A*' (and only these elements) are the solutions of Problem (6) with different types of trade-offs represented by inequalities. The maximum fitness that the colony can reach (i.e., the optimal fitness that the colony can reach by choosing any strategy from *A*') can be calculated using Eq (8).

*Case 3*'. This case implies that the resource constraint influences the colony’s well-being and that the set *A*' is empty. Here, the mathematical solution becomes more complex and depends on the parameters of the model (see Appendix C in S1 Appendix). If all trade-off functions are concave or linear, then the set of optimal strategies can be found as follows. We should find strategies that maximize or minimize (depending of the model's parameters) the total fecundity of the colony subject to all trade-off constraints and under the assumption that all available resources are consumed by the colony (i.e., subject to the resource constraint, assuming that this constraint is binding).

Let us now consider Problem (6) with different types of trade-off functions (i.e., *Original problem*). We wonder how the solutions of Original problem change after replacing equalities by inequalities in trade-offs (after these changes we get Problem (6) with different types of trade-off functions and trade-offs inequalities, i.e., *Modified problem*).

- First, we assume that initially we had Case 1. The change of equalities by inequalities in the trade-off constraints does not change the problem's solutions. After this change, we get Case 1' in Modified problem. Indeed, assume that the strategy (b*,v*) is a solution of Original problem. This strategy remains feasible in the corresponding Modified problem. Let us now consider the strategy (*b*,*v*) in Modified problem, such that there exists a subset of cells, *M*, in the colony, such that for any cell *i* of *M*, we have: *v*_*i*_ < *φ*_*i*_(*b*_*i*_). The strategy (*b*,*v*) is not optimal. To prove this statement, we consider a strategy (*b*^1^,*v*^1^), such that *b*_*i*_ = *b*_*i*_^1^ for each cell *i* of the colony, and *v*_*i*_ = *v*_*i*_^1^ for each cell *i* that is not in *M*, and *v*_*i*_^1^ = *φ*_*i*_(*b*_*i*_) for each cell *i* in *M*. We can see that *B*(*b*,*v*) = *B*(*b*^1^,*v*^1^) and *V*(*b*,*v*) < *V*(*b*^1^,*v*^1^), and so *W*(*b*, *v*) < *W*(*b*^1^, *v*^1^). If the strategy (*b*^1^,*v*^1^) is in the domain of Modified problem, the statement is proved. If not, we have to prove that *W*(*b*^1^,*v*^1^) ≤ *W*(b*,v*). Indeed, let us consider the strategy (b*,v*)–a solution of Original problem in Case 1. It means that (b*,v*) is a solution of Original problem in the absence of the resource constraint. Because (*b*^1^,*v*^1^) belongs to the domain of this problem, we can conclude that *W*(*b*^1^, *v*^1^) ≤ *W*(b*, v*). Taking into account that the strategy (b*,v*) is feasible in Modified problem, we can also conclude that (*b*,*v*) is not optimal. Therefore, the solution of Modified problem belongs to the domain of Original problem. Furthermore, the domain of Original problem is a subset of the domain of Modified problem. Thus, we conclude that (b*,v*) is a solution of Modified problem in Case 1'.

- Second, we assume that initially we had Case 2. Thus, the set *A* represents the solution of Original problem. After changing the trade-off constraints (from equalities to inequalities), all points of the set *A* becomes points of the set *A*'. It means that we get Case 2' in Modified problem and all solutions of Original problem become solutions of Modified problem. More precisely, the set *A*' of all solutions of Modified problem contains all strategies from *A* as well as possible additional solutions, (b*,v*), for which some trade-off constraints are not binding.

- Third, we assume that initially we had Case 3. It means that the resource constraint matters and the set *A* is empty. As mentioned above, after changing the trade-off constraints (from “=“ to “≤”), all points of *A* becomes points of *A*'. Even though the set *A* is empty, the set *A*' can be nonempty. It leads to two different cases. The first case implies that the set *A*' is nonempty, and we get Case 2' of Modified problem. Here all optimal strategies require the existence of at least one cell in the colony, such that viability of this cell is smaller than the maximal possible viability that this cell can attain for a fixed level of fecundity (under the assumption that the only constraint that matters is the trade-off constraint). The second case implies that the set *A*' is empty. It means that we get Case 3' of Modified problem.

To complete our analysis, we consider the case of an empty domain in Original problem (denoted here as Case 0). Assume that we have an environment, such that the domain in Original problem is empty. After changing the trade-off constraints (from equalities to inequalities), we get Modified problem. In this case, we state that we get Case 2 in Modified problem. To prove this statement, we need to show that the set *A*' in Modified problem is non-empty. Because we are in Case 0 in Original problem, $k_{1}\sum_{i=1}^{N}b_{i}^{\max}>C\geq k_{1}\cdot \frac{\alpha \cdot C}{k_{1}(\alpha +\beta )}$, and thus $\sum_{i=1}^{N}b_{i}^{\max}>\frac{\alpha \cdot C}{k_{1}(\alpha +\beta )}$. Therefore, there exists *b* from *R*^*N*^, such that $0\leq b_{i}\leq b_{i}^{\max}$ for all *i* and $\sum_{i=1}^{N}b_{i}=\frac{\alpha \cdot C}{k_{1}(\alpha +\beta )}$. Because we are in Case 0, we have $k_{1}\sum_{i=1}^{N}b_{i}+k_{2}\sum_{i=1}^{N}\varphi_{i}(b_{i})>C$. Because $\sum_{i=1}^{N}b_{i}=\frac{\alpha \cdot C}{k_{1}(\alpha +\beta )}$ and $k_{1}\times \frac{\alpha \cdot C}{k_{1}(\alpha +\beta )}+k_{2}\times \frac{\beta \cdot C}{k_{2}(\alpha +\beta )}=C$, we get: $\sum_{i=1}^{N}\varphi_{i}(b_{i})>\frac{\beta \cdot C}{k_{2}(\alpha +\beta )}$. Therefore, there exists *v* from *R*^*N*^ such that 0≤*v*_*i*_≤*φ*_i_(*b*_i_) for all *i* and $\sum_{i=1}^{N}v_{i}=\frac{\beta \cdot C}{k_{2}(\alpha +\beta )}$. It means that the point (*b*, *v*) is in *A*'. Thus, we are in Case 2 of Modified problem.

Case 0 can occur here only with equalities in the trade-off relationships. This case arises with a very low level of available resources and implies that the colony has no feasible strategies. In other words, if the environment is very poor, the colony dies in this environment. It looks reasonable from a biological point of view, that there exists a certain resource threshold, *C*^#^, such that if the amount of resources available to the colony becomes smaller than *C*^#^, then the colony dies. In our opinion, the existence of Case 0 is an advantage of the model with trade-offs in the form of equalities. Conversely, for each positive value of *C*, there is a feasible strategy for the colony in the model with trade-offs in the form of inequalities. Of course, the fitness of the colony tends to zero as the quality of environmental conditions tends to zero. So, formally, the colony has a feasible strategy and a positive, but a very low, fitness even though the amount of available resources is close to zero, i.e., the colony can survive in such an environment. Therefore, we should artificially introduce the threshold value of fitness, *W*^#^, into the model with trade-offs in the form of inequalities, such that the colony dies in an environment in which the highest attainable fitness is smaller than *W*^#^. Thus, we showed that the replacement of the trade-off functions *v*_*i*_ = *φ*_*i*_(*b*_*i*_) by *v*_*i*_ < *φ*_*i*_(*b*_*i*_) leads to a different analysis in Cases 3 and 0. It is worth noting that these results hold as well for non-additive forms of the viability function.

In addition, we will show that for Problem (6) with different types of trade-offs represented by inequalities, there is a direct link between the quality of the environment and the Case of solution obtained for this environment. Precisely, Case 1' occurs with large values of *C*, Case 2' with small values of *C*, and Case 3', if any, with intermediate values of *C*. This conclusion stems from the two following observations:

- Let us consider Problem (6) with different types of trade-offs represented by inequalities, but without the resource constraint. We will determine the set *S* of all solutions of this problem. This set is compact, so we can identify the minimal possible value, C*, of *С*, such that there exists a solution (b*,v*) in *S* satisfying the following inequality: $k_{1}\sum_{i=1}^{N}b_{i}^{*}+k_{2}\sum_{i=1}^{N}v_{i}^{*}\leq C^{*}$. Let us now consider Problem (6) with different types of trade-offs represented by inequalities and the resource constraint. For all *C* ≥C*, Case 1' should occur here.

- Let us assume that there exists a level of available resources, C^**^ > 0, such that the set *A*'(C^**^) is nonempty. It means that Case 2' occurs in Problem (6) with different types of trade-offs represented by inequalities when C^**^ is the amount of available resources. Let (b^**^,v^**^) be a solution belonging to *A*'(C^**^). We need to show that for all positive *C* < C^**^, Case 2' occurs in the considered version of Problem (6). In other words, we have to prove that *A*'(*C*) is nonempty for all positive *C* < C^**^. First of all, that each value of *σ*, such that 0 ≤ *σ* ≤ C^**^, can be represented as *σ* = λC^**^, where 0 ≤ *λ* ≤ 1. We need to show that the strategy (λb^**^,λv^**^) belongs to *A*'(*σ*). Indeed, *λb*_i_^**^ ≥ 0 and *λv*_i_^**^ ≥ 0 for all cells of a given colony, and $\sum_{i=1}^{N}\lambda b_{i}{}^{**}=\frac{\alpha \sigma }{k_{1}\left(\alpha +\beta \right)}\;\text{and}\;\sum_{i=1}^{N}\lambda v_{i}{}^{**}=\frac{\beta \sigma }{k_{2}\left(\alpha +\beta \right)}$. Moreover, for all cells of the colony we also have: $\lambda v_{i}{}^{**}\leq v_{i}{}^{**}\leq \varphi_{i}\left(b_{i}^{**}\right)\leq \varphi_{i}\left(\lambda b_{i}{}^{**}\right),$ where the last inequality results from the assumption of a strictly decreasing form of trade-off functions. Thus, we showed that for the trade-off relationships of the form *v*_*i*_ ≤ *φ*_*i*_(*b*_*i*_), Case 3' takes place in environments of intermediate quality, Case 1' in environments of good quality, and Case 2' in environments of bad quality. In general, this is not true for the trade-off relationships of the form *v*_*i*_ = *φ*_*i*_(*b*_*i*_). Here, Case 1 takes place in environments of good quality too, and Case 0 in environments of bad quality, but the situation with Cases 2 and 3 is more sophisticated. To show this, we consider the following numerical example which involves three cells with the following trade-off functions: *v*_1_ = 6 – 3*b*_1_^2^, *v*_2_ = 4 –*b*_2_^2^ and *v*_3_ = 5 – 2*b*_3_^2^. These trade-off functions are concave because we consider a small-sized colony. Let *α* = *β* = 1, *k*_1_ = 2 and *k*_2_ = 3. We will vary the level of available resources, *C*, in order to determine the Case of solution corresponding to it. First of all, assume that all trade-off relationships have the form of inequalities. For all values of *C* that are larger than *C*_1_ (*C*_1_ ≈ 36.055), Case 1' takes place, so all cells are unspecialized. For all values of *C* that are smaller than *C*_1_ and larger than *C*_2_ (*C*_2_ ≈ 18.3), Case 3' takes place. Here, the solution of the problem is a single point. Moreover, for all values of *C* that are smaller than *C*_3_ (*C*_3_ ≈ 30.3) and larger than *C*_2_, the optimal strategy implies that the second cell specializes in reproductive function. For all values of *C* that are slightly larger than *C*_2_, the optimal strategy implies that *b*_3_* ≈ *b*_3_^max^, i.e., we can say that at the optimum, cells 2 and 3 should specialize in reproductive function, while cell 1 remains unspecialized. For all positive values of *C* that are smaller than *C*_2_, Case 2' takes place and the solution represents a set of states. Furthermore, the decrease in the value of *C* directs the colony to unspecialized states. Thus, this example confirms that Case 3' takes place in environments of intermediate quality, Case 1' in environments of good quality and Case 2' in environments of bad quality when trade-offs are represented by inequalities. Now we replace now inequalities by equalities in trade-offs. Following our theory, for all values of *C* that are larger than *C*_1_, Case 1 takes place and for all values of *C* that are smaller than *C*_1_ and larger than *C*_2_, Case 3 takes place. Moreover, for these values of *C*, the solution does not change. However, for the values of *C* that are smaller than *C*_2_, some changes occur. Precisely, for all values of *C* that are smaller than *C*_2_ and larger than *C*_4_ (*C*_4_ ≈ 16.5), Case 2 with unspecialized optimal states takes place, but for all values of *C* that are smaller than *C*_4_ and larger than *C*_5_ (*C*_5_ ≈ 9.9), Case 3 occurs again. In the latter situation, cells 1 and 3 specialize in germ, while cell 2 remains unspecialized. For all values of *C* that are smaller than *C*_5_, Case 0 takes place. It means that the amount of resources available to the colony is smaller than the amount of resources sufficient for the colony’s existence.

In this example, considering trade-offs in the form of inequalities, specialization occurs when the resource constraint influences the colony’s well-being, but the amount of available resources is rather high. For trade-offs in the form of equalities, specialization occurs when the resource constraint influences the colony’s well-being, whereas the amount of available resources can be rather high or rather small, but sufficient for the colony’s survival. This case arises because the resource constraint prohibits the second cell from being specialized in germ. Thus, the second cell remains unspecialized. Moreover, in this example fecundity is ‘cheaper’ than viability. Consequently, the first and the third cells should be specialized in germ. If we replace trade-offs in the form of equalities by those in the form of inequalities, each cell would have the opportunity to reduce its viability when its fecundity is fixed. Thus, in this case, the colony will be able to choose strategies that yield better fitness, and the unspecialized strategy becomes optimal.

## Discussion

We have presented and analyzed a new general mathematical model for studying the emergence of germ-soma specialization. We have examined how the division of labor in a cell colony depends on the shape of trade-off functions, a resource constraint and different fecundity and viability rates. Thus, here we have generalized the popular fitness model introduced by Michod et al. [14]. We have described the set of all possible solutions of the formulated mathematical programming problem and have depicted some interesting examples of optimal solutions found using our fitness function. We have shown that the changes in structural complexity in unspecialized large-sized colonies can lead to the emergence of specialization as well as to unspecialized optimal states in the case of environmental quality degradation (i.e., the lack of available environmental resources). This means that the process of functional specialization of Volvocalean green algae (GS–GS/S) can be explained not only in terms of increasing the colony size [15]. Moreover, we argue that significant resource restrictions and a trade-off between the production difficulties and the fitness benefits of fecundity can cause the emergence of specialization in small-sized colonies. We have also considered the case of different trade-off functions for different cells of the colony. Thus, we have assumed that some cells of the colony may not be equivalent with respect to viability or fecundity. We have explored several factors that can lead to the emergence of this non-equivalence, such as mutations, a specific developmental program, and positional effects. We have shown that positional effects and mutations in small-sized colonies can lead to the emergence of soma specialization without changes in size or in resource restrictions.

To conclude, we have described some cases in which the colony specialization can emerge. The existence of these cases is supported by a variety of experimental work [13], [26] and [27], but it would be certainly reasonable to provide more detailed quantitative analysis for testing the predictions of our models. Thus, it would be interesting to test our model in practice. One possible test of our model is to evolve Volvocalean green algae (small-sized and large-sized colonies) under different resource constraints and environmental conditions. Moreover, in this kind of experiment we should keep in mind that the generation time, or the time for growth, can change during the evolutionary process, and take it into account in the model. It is worth noting that different positional effects can occur during the experiment. Consequently, we should divide cells into different relevant groups. Such a division will allow testing the model with different types of cells.

Another interesting issue is the model's specification. In particular, this issue concerns the form of trade-off relationships. We have considered two versions of our model: with trade-offs of the form *v*_*i*_ = *φ*_*i*_(*b*_*i*_) and those of the form *v*_*i*_ ≤ *φ*_*i*_ (*b*_*i*_). In the previous section, we have described all possible situations where the solutions of the related problems have the same structure, or even coincide, as well as where they have different structures. We have discussed different specialization effects that can take place in different instances of our model. Clearly, focusing on a specific type of organisms would allow a better understanding of the true specialization effects and, consequently, would indicate the best way of modeling the trade-off constraints. Moreover, it is worth noting that clarifying the best way of modeling the environmental impact is an essential issue as well. Here, we have considered the resource constraint of the form (5). However, some questions concerning the parameters of this constraint remain open. For example, should the cost, in terms of resources necessary to produce a unit of fecundity or a unit of viability, depend on the size of the colony, i.e., should the parameters *k*_1_ and *k*_2_ be functions of *N*? Or, should the cost, in terms of resources necessary to produce a unit of fecundity, be the same when this fecundity is produced by a single germ cell or two generalist cells, i.e., should the parameter *k*_1_ be a function of *B* and *V*? Or, should the quantity of resources necessary to produce a unit of viability depend on positional effects, i.e., should the parameter *k*_2_ be a function of the variables *v*_1_,..,*v*_*N*_? There are also some interesting questions concerning the best way of modeling the effect of a limiting environment. For example, instead of the resource constraint of the form (5), one could consider a parameterized family of trade-off functions, such that *v*_*i*_ = *φ*_*i*_(*b*_*i*_,*C*) (see for example [14] or [32]). Such a model would be different from that described in our paper, though possibly, some equivalence between the two models could be found at some optimal states. One important advantage of the resource constraint of the form (5) is that all parameters of this constraint can be measured directly. In the case of a parameterized family, *v*_*i*_ = *φ*_*i*_(*b*_*i*_,*C*), the parameter estimation process seems to be much more complicated.

Another important issue is the robustness of our model. On one hand, our model is robust because its qualitative results do not depend on the specific form of trade-off functions. They depend only on the curvature of these functions (i.e., convex, linear or concave), which is determined by the size of the colony. On the other hand, we have mentioned that the assumption of additivity is rather questionable for the viability function. We have shown that in many instances of our model the property of additivity is not necessary for the viability functions. However, some of our results are based on the additivity assumption. It would be interesting to investigate how these results would change in the case of a non-additive form of the viability function. For example in this work, as well as in the studies of Michod et al. and Solari et al. [14–15], it is assumed that all cells of the colony have trade-offs with the same type of curvature. It would be also interesting to analyze the case where some cells of the colony have convex trade-offs and the other have concave trade-offs. According to a recent study of Leslie et al. [33], it is natural to assume that such a case can occur in an intermediate-sized colony in which we have cell generation times such that the colony's trade-off functions are linear, on average, but due to within-colony variation some of them can be slightly convex or concave. Moreover, some cells can have neither convex nor concave trade-off functions that correspond to intermediate initial costs of reproduction [14]. It is clear that in such a situation Case 2 described here does not change, but the analysis of Cases 1 and 3 becomes more complicated because some additional parameters, including the numbers of “convex” and “concave” cells in the colony, will be added to the model. An extensive additional analysis would be required to find optimal specialization patterns in these instances. If both convex and concave trade-offs are allowed in the model, Case 1 of this model should represent a kind of average between Case 1 in the strictly convex and Case 1 in the strictly concave trade-off models in terms of the number of specialized cells in the colony. Also, we can anticipate that the general principle of finding solutions in Case 3 with concave and linear trade-offs would not change here: the optimal reproductive strategies would be the available strategies from the resource surface such that they have the same total fecundity and the value of this total fecundity is the closest one (among all available strategies from the resource surface) to the unavailable total fecundity calculated according to Eq (7). Nevertheless, the detailed structure and the geometry of these solutions seem to be much more diverse and complex than the solution structure of Case 3 presented in this paper.

Another challenging question is the investigation of the role of positional effects in our model. We can incorporate into the model the information about the form of the colony (e.g., sphere or line) and consider the impact of positional effects in each case. Such a development will enable us to gain more insight into the structure and properties of trade-off functions.

It is worth noting that the proposed model can be generalized to address a number of relevant biological issues, including the evolution of specialized enzymes or the emergence of complex organs. Importantly, the presented fitness model is of a general nature and could be also applied to address a number of parallel issues in different fields, such as economics, where fitness could be associated with the production volume, or education, where fitness could be associated with the effectiveness of the process of education, or health care, where fitness could be associated with life expectancy or life quality.

## Supporting information

S1 Appendix The proofs of main results. (DOCX)
