# Supplementary material for: Modeling functional specialization of a cell colony under different fecundity and viability rates and resource constraint
Source: PLoS One. 2018 Aug 8;13(8):e0201446. doi: 10.1371/journal.pone.0201446 (PMC6082568; doi:10.1371/journal.pone.0201446)
Supplement: S1 Appendix — (DOCX) [file pone.0201446.s001.docx]

## Appendix A

Consider a colony of *N* cells, with cells indexed *i* = 1, .., *N*. Let *b_i_* be fecundity of the cell *i* and *v_i_* be viability of the cell *i*. Denote *b =* (*b*_1_,..*,b_N_*) and *v =* (*v*_1_,..,*v_N_*). Let *W* be the fitness function of the colony. Here, *φ_i_*(*b_i_*), *i*=1,…, *N*, are intrinsic trade-off functions of cells. The parameters *α* and *β* reflect the “importance” of fecundity and viability contributions to the fitness of the colony, respectively (*α* and *β* > 0). The parameter *C* represents the amount of resources available to the colony. The parameter *k*_1_ represents the amount of resources necessary to produce one unit of fecundity and the parameter *k*_2_ represents the amount of resources necessary to produce one unit of viability, *k*_1_ > 0, *k*_2_ > 0 and *C* > 0. Consider the following optimization problem:

$$\left\{ \begin{aligned} W=\left( \sum_{i=1}^{N} b_{i} \right)^{\frac{\alpha}{\beta}}\sum_{i=1}^{N} \varphi_{i}\left( b_{i} \right)\to{max}_{b} \\ \text{for all }i=\bar{1,N}: 0\leq b_{i}\leq b_{i}^{max}. \end{aligned} \right.,$$

(A1)

This problem is equivalent to Problem (6) with different types of trade-off functions in the form of equalities, and without the resource constraint (5), since the function $f\left( W \right)=W^{\beta}$ is a monotonic transformation of the function *W*.

Let $H\text{ be the set of all }b\in R^{N}\text{, such that }{0\leq b}_{i}\leq b_{i}^{max} \text{for all }i=\bar{1,N}$ – the domain of the problem under study. Consider a set of triples$\left( I_{1}^{p},I_{2}^{p},I_{3}^{p} \right)$, such that$I_{1}^{p}\subset\left\{ 1,..,N \right\};I_{2}^{p}\subset\left\{ 1,..,N \right\}; I_{3}^{p}\subseteq\left\{ 1,..,N \right\}$,$I_{1}^{p}\cup I_{2}^{p}\cup I_{3}^{p}=\left\{ 1,..,N \right\}$,$I_{1}^{p}\cap I_{2}^{p}=I_{1}^{p}\cap I_{3}^{p}=I_{2}^{p}\cap I_{3}^{p}=\emptyset$ and$\left| I_{3}^{p} \right|\geq1$. For the sake of simplicity, we have indexed each triple by a parameter $p$ from some set$\tilde{\mathcal{P}}$, such that there is a bijection (given by the indexation) between the set$\tilde{\mathcal{P}}$ and the set of triples. Let $H_{p}$ be the set of all $b\in R^{N}$, such that ${0\leq b}_{i}\leq b_{i}^{max}$ for all $i\in I_{3}^{p};$ $b_{i}=b_{i}^{max}$ for all $i\in I_{1}^{p};$ $b_{i}=0$ for all $i\in I_{2}^{p}$. Each set $H_{p}$ represent a face (or an edge) of hyperparallelepiped *H*. In particular, consider the element *p* of $\tilde{\mathcal{P,}}$ indexed by the triple$\left( \emptyset, \emptyset, \left\{ 1,..,N \right\} \right)$. The corresponding set $H_{p}$ is the whole set *H*. Consider $b\in R^{N}$. Let $U_{\varepsilon}\left( b \right)$ be an open ball, where $\varepsilon>0$, and consider the set of all $\tilde{b}\in R^{N}$, such that $\left\| b-\tilde{b} \right\|<\varepsilon$. We will say that point $b\in H_{p}$ is a point of local maximum of the function *W* on $H_{p}$ if and only if there exists an open ball $U_{\varepsilon}\left( b \right)$, such that for all $\tilde{b}\in U_{\varepsilon}\left( b \right)\cap H_{p}$, the following inequality holds: $W(b)\geq W(\tilde{b})$. We will say that point $b\in H_{p}$ is a point of global maximum of the function *W* on $H_{p}$ if and only if for all $\tilde{b}\in H_{p}$, the following inequality holds: $W(b)\geq W(\tilde{b})$.

**Proposition 1**

Let all functions $\varphi_{i}\left( b_{i} \right)$ be strictly concave. Consider some$p\in\tilde{\mathcal{P}}$ and a point$b_{p}^{1}\in H_{p}$, such that $b_{p}^{1}$ is a point of local maximum of the function *W* on $H_{p}$. Then, there are no points $b_{p}^{0}\in H_{p}$ such that $b_{p}^{0}\neq b_{p}^{1}$ and $W(b_{p}^{0})\geq W(b_{p}^{1})$. Consequently, we can conclude that for each $p\in\tilde{\mathcal{P}}$ there exists the unique point $b_{p}^{*}\in H_{p}$, such that this point is the point of local (and global) maximum of the function *W* on $H_{p}$. Moreover, assume that for some $p\in\tilde{\mathcal{P}}$ there exists a point $b_{p}\in{ReInt(H}_{p}),$ such that $\frac{d\varphi_{i}}{db_{i}}\left( b_{pi} \right)=-\frac{\alpha V\left( b_{p} \right)}{\beta B\left( b_{p} \right)}$ for all $i\in I_{3}^{p}$. Then, this and only this point is the point of global maximum of the function *W* on $H_{p}$.

Before proving it, let us briefly discuss and interpret some conclusions which can be drawn from Proposition 1. First, Proposition 1 tells us that the point of local maximum of the function *W* on *H* is unique, and that this point is the unique solution of Problem (A1). Moreover, if there exists a point $b\in Int(H),$ such that $\frac{d\varphi_{i}}{db_{i}}\left( b_{i} \right)=-\frac{\alpha V\left( b \right)}{\beta B\left( b \right)}$ for all $i\in\{1,..,N\}$, this point would be the unique solution of Problem (A1). Second, this proposition provides a necessary and sufficient condition for the emergence of specialization in small-sized colonies. In small-sized colonies, cell specialization arises if and only if there are no points *b* from the interior of the set *H*, such that $\frac{d\varphi_{i}}{db_{i}}\left( b_{i} \right)=-\frac{\alpha V\left( b \right)}{\beta B\left( b \right)}$ for all $i\in\{1,..,N\}$. Third, assume that for several reasons some cells of the colony are specialized. Proposition 1 postulates that other cells remain unspecialized if and only if there exists the reproductive strategy *b*, such that $\frac{d\varphi_{i}}{db_{i}}\left( b_{i} \right)=-\frac{\alpha V\left( b \right)}{\beta B\left( b \right)}$ for each undecided cell *i*. Also, note that Proposition 1 reveals a very specific geometry of Problem (A1), such that the solution of this problem can be found easily in practice.

**Proof**

Choose and fix some $p\in\tilde{\mathcal{P}}$. Let $b_{p}^{1}$ represents a point of local maximum of the function *W* on $H_{p}$, i.e., there exists an open ball $U_{\varepsilon}\left( b_{p}^{1} \right), \text{where }\varepsilon>0$, such that for all $\tilde{b_{p}}\in U_{\varepsilon}\left( b_{p}^{1} \right)\cap H_{p}$, the following inequality holds: $W(b_{p}^{1})\geq W(\tilde{b_{p}})$. We should prove that there are no points $b_{p}^{0}\in H_{p}$, such that $b_{p}^{0}\neq b_{p}^{1}$ and $W(b_{p}^{0})\geq W(b_{p}^{1})$.

To prove this statement, we first note that the function *V*(*b*) is strictly concave. Second, we provide the following auxiliary statement: Consider two points *b*^'^ and *b*^''^ from *H*, such that *b*^'^ ≠ *b*^''^ and *W*(*b*^'^) = *W*(*b*^''^) > 0. Then, for all ξ from (0, 1) the following inequality holds: *W*(ξ*b*^'^ + (1 - ξ) *b*^''^) > *W*(*b*^'^). We denote this statement as Lemma.

To prove this Lemma, consider a function *W*^#^(*b*) = *f*(*b*)*∙g*(*b*). If $\alpha\leq\beta$, we assume that *f*(*b*) = $B^{\frac{\alpha}{\beta}}(b)$ and *g*(*b*) = *V*(*b*). If $\alpha>\beta$, we assume that *f*(*b*) = *B*(*b*) and *g*(*b*) = $V^{\frac{\beta}{\alpha}}(b)$. We can conclude that the function *f* is concave and the function *g* is strictly concave. Moreover, $W^{\#}\left( b \right)=W(b)$, if $\alpha\leq\beta$ and $W^{\#}\left( b \right)=W^{\frac{\beta}{\alpha}}(b)$, if $\alpha>\beta$. It means that for all points $b$ and $\tilde{b}$ from *H*, *W*($b$) > *W*($\tilde{b}$) if and only if *W*^#^($b$) > *W*^#^($\tilde{b}$), and *W*($b$) = *W*($\tilde{b}$) if and only if *W*^#^($b$) = *W*^#^($\tilde{b}$). We know that *W*(*b*^'^) = *W*(*b*^''^). Consequently, *W*^#^(*b*^'^) = *W*^#^(*b*^''^), i.e., *f*(*b*^'^) g(*b*^'^) = *f*(*b*^''^) g(*b*^''^). Moreover, this means that *f*(*b*^'^) ≥ *f*(*b*^''^) if and only if *g*(*b*^''^) ≥ *g*(*b*^'^). Then, we have the following estimation: *W*^#^(ξ*b*^'^ + (1 - ξ) *b*^''^) = *f*(ξ*b*^'^ + (1 - ξ) *b*^''^) *g*(ξ*b*^'^ + (1 - ξ) *b*^''^) > (ξ*f*(*b*^'^) + (1 - ξ) *f* (*b*^''^))*∙*(ξg(*b*^'^) + (1 - ξ) *g*(*b*^''^)) = *f*(*b*^'^) g(*b*^'^) + ξ (1 - ξ)*∙*(*f*(*b*^'^) - *f*(*b*^''^)) (*g*(*b*^''^) - *g*(*b*^'^)) ≥ *f*(*b*^'^) *g*(*b*^'^) = *W*^#^(*b*^'^). Consequently, *W*(ξ*b*^'^ + (1 - ξ) *b*^''^) > *W*(*b*^'^). Thus, Lemma is proved.

Now assume that there exists a point $b_{p}^{0}$ from *H_p_*, such that $b_{p}^{0}\neq b_{p}^{1}$ and $W(b_{p}^{0})\geq W(b_{p}^{1})$. Consider the set $\Omega_{1}$ of all points$b_{p}^{\mu}\in R^{N}$, such that$b_{p}^{\mu}=\mu b_{p}^{1}+\left( 1-\mu\right)b_{p}^{0}, \mathrm{where} 0\leq\mu\leq1$. Since $b_{p}^{0}$ and $b_{p}^{1}$ belong to *H_p_* and *H_p_* is convex, we can conclude that $\Omega_{1}\subset H_{p}$. Denote $\tilde{\mu}=max\left\{ 0; 1-\frac{\varepsilon}{2\left\| b_{p}^{0}-b_{p}^{1} \right\|} \right\}$. For all $\mu\in\left[ \tilde{\mu}, 1 \right],$ $b_{p}^{\mu}$ belongs to the set $U_{\varepsilon}\left( b_{p}^{1} \right)\cap\Omega_{1}\subset U_{\varepsilon}\left( b_{p}^{1} \right)\cap H_{p}$, i.e., $W(b_{p}^{1})\geq W(b_{p}^{\mu})$ for all $b_{p}^{\mu}, \mu\in\left[ \tilde{\mu}, 1 \right]$. Consider the behavior of the function *W* on the set $\Omega_{1}$. Two cases can take place here. In the first case, $W\left( b_{p}^{0} \right)=W\left( b_{p}^{1} \right)$. According to Lemma above, we can conclude that for all $b_{p}^{\mu}, such that \mu\in(0, 1)$, the following inequality holds: $W\left( b_{p}^{\mu} \right)> W(b_{p}^{1})$. Consequently, we reached a contradiction. In the second case, $W\left( b_{p}^{0} \right)>W\left( b_{p}^{1} \right)$. Consider a closed interval $\left[ b_{p}^{\tilde{\mu}}, b_{p}^{0} \right]$. Using the fact that $W\left( b_{p}^{0} \right)>W\left( b_{p}^{1} \right)$ and $W\left( b_{p}^{1} \right)\geq W(b_{p}^{\tilde{\mu}})$, we can apply the intermediate value theorem and conclude that there exists $\tilde{\tilde{\mu}}\in(0,\tilde{\mu}]$, such that $W\left( b_{p}^{1} \right)=W(b_{p}^{\tilde{\tilde{\mu}}})$. According to Lemma above, we can conclude that for all $b_{p}^{\mu}, such that \mu\in(\tilde{\tilde{\mu}}, 1)$, the following inequality holds: $W\left( b_{p}^{\mu} \right)> W(b_{p}^{1})$. Thus, we reach a contradiction, and the first part of Proposition 1 is proved.

Now, fix some$value of p\in\tilde{\mathcal{P}}$. Let $b_{p}^{*}\in{ReInt(H}_{p})$be a stationary point of the function $W$ on $H_{p}$. Therefore, $b_{p}^{*}$ should satisfy the following equation [1]:

$$\nabla W\left( b_{p}^{*} \right)=0\text{ if and only if for all }i \text{and }j\in I_{3}^{p}:\frac{d\varphi_{i}}{db_{pi}}\left( b_{pi}^{*} \right)=\frac{d\varphi_{j}}{db_{pj}}\left( b_{pj}^{*} \right)=-\frac{\alpha V\left( b_{p}^{*} \right)}{\beta B\left( b_{p}^{*} \right)}.$$

(A2)

Denote $\varphi^{'}=\frac{d\varphi_{i}}{db_{pi}}\left( b_{pi}^{*} \right)=\frac{d\varphi_{j}}{db_{pj}}\left( b_{pj}^{*} \right), \text{for all }i,j\in I_{3}^{p}$.

In order to study the behavior of the function $W$ in the stationary point $b_{p}^{*}$, we will use the second order conditions [2]. First, calculate the second derivatives of $W$:

$$\frac{\partial^{2}W}{\partial b_{pi}^{2}}\left( b_{p}^{*} \right)=B^{\frac{\alpha}{\beta}-1}\left( b_{p}^{*} \right)\left[ \left( \frac{\alpha}{\beta}+1 \right)\varphi^{'}+B\left( b_{p}^{*} \right)\frac{d^{2}\varphi_{i}}{db_{pi}^{2}}\left( b_{pi}^{*} \right) \right], \text{for all }i\in I_{3}^{p},$$

$$\frac{\partial^{2}W}{\partial b_{pi}\partial b_{pj}}\left( b_{p}^{*} \right)=\left( \frac{\alpha}{\beta}+1 \right)\varphi^{'}B^{\frac{\alpha}{\beta}-1}\left( b_{p}^{*} \right), \text{for all }i\neq j\in I_{3}^{p}.$$

Let us denote:

$$\psi_{i}=\frac{d^{2}\varphi_{i}}{db_{pi}^{2}}\left( b_{pi}^{*} \right), \text{for all }i\in I_{3}^{p}, \theta=\left( \frac{\alpha}{\beta}+1 \right)\varphi^{'}, B\left( b_{p}^{*} \right)=Z,$$

$$t_{1}^{p}=\min\{i, \text{such that }i\in I_{3}^{p}\} \text{and }t_{d}^{p}=\min\{i, \text{such that }i\in I_{3}^{p}\backslash\bigcup_{f=1}^{d-1} \{t_{f}^{p}\}\}, \text{for all }d\in\{2,..,\left| I_{3}^{p} \right|\}.$$

Thus, we can represent the Hesse matrix, calculated in the point $b_{p}^{*}$, as follows [3]:

$$He\left( p,b_{p}^{*} \right)=Z^{\frac{\alpha}{\beta}-1}\left( \begin{matrix} {Z\psi}_{t_{1}^{p}}+\theta& \theta& \begin{matrix} \ldots& \theta\end{matrix} \\ \begin{matrix} \theta\\ \vdots\end{matrix} & \begin{matrix} Z\psi_{t_{2}^{p}}+\theta\\ \vdots\end{matrix} & \begin{matrix} \begin{matrix} \ldots& \theta\end{matrix} \\ \begin{matrix} \ddots& \vdots\end{matrix} \end{matrix} \\ \theta& \theta& \begin{matrix} \ldots& {Z\psi}_{t_{\left| I_{3}^{p} \right|}^{p}}+\theta\end{matrix} \end{matrix} \right).$$

Consider the following determinant ($1\leq n\leq\left| I_{3}^{p} \right|)$:

$I\left( n \right)=Z^{n(\frac{\alpha}{\beta}-1)}\left| \begin{matrix} {Z\psi}_{t_{1}^{p}}+\theta& \theta& \begin{matrix} \ldots& \theta\end{matrix} \\ \begin{matrix} \theta\\ \vdots\end{matrix} & \begin{matrix} Z\psi_{t_{2}^{p}}+\theta\\ \vdots\end{matrix} & \begin{matrix} \begin{matrix} \ldots& \theta\end{matrix} \\ \begin{matrix} \ddots& \vdots\end{matrix} \end{matrix} \\ \theta& \theta& \begin{matrix} \ldots& {Z\psi}_{t_{n}^{p}}+\theta\end{matrix} \end{matrix} \right|$.

Then, $I\left( n \right)=Z^{n\frac{\alpha}{\beta}-1}\left[ Z\prod_{i=1}^{n} \psi_{t_{i}^{p}}+\theta\sum_{k\in K} \prod_{i\in k} \psi_{t_{i}^{p}} \right]$, where $K \text{is the set of all }k\in2^{\left\{ 1,..,n \right\}}$, such that $\left| k \right|=n-1$. To prove this fact, first of all denote:

$$a_{i}=\left( \begin{matrix} \begin{matrix} 0 & \ldots\end{matrix} & Z\psi_{t_{i}^{p}} & \begin{matrix} \ldots& 0 \end{matrix} \end{matrix} \right)^{T}, \text{for all }i=1,..,n \text{and} \Theta=\left( \begin{matrix} \begin{matrix} \theta, & \ldots\end{matrix} & \theta, & \begin{matrix} \ldots& \theta\end{matrix} \end{matrix} \right)^{T}.$$

Now we can calculate the determinant *I*(*n*) as follows:

$$\frac{I\left( n \right)}{Z^{n(\frac{\alpha}{\beta}-1)}}=\left| a_{1}+\Theta;a_{2}+\Theta;\ldots;a_{n}+\Theta\right|=\left| a_{1};a_{2}+\Theta;\ldots;a_{n}+\Theta\right|+\left| \Theta;a_{2}+\Theta;\ldots;a_{n}+\Theta\right|,$$

$$\frac{I\left( n \right)}{Z^{n(\frac{\alpha}{\beta}-1)}}=\left| a_{1};a_{2};\ldots;a_{n}+\Theta\right|+\left| a_{1};\Theta;\ldots;a_{n} \right|+\left| \Theta;a_{2};\ldots;a_{n} \right|,$$

$$\frac{I\left( n \right)}{Z^{n(\frac{\alpha}{\beta}-1)}}=\left| a_{1};a_{2};\ldots;a_{n} \right|+\left| \Theta;a_{2};\ldots;a_{n} \right|+\left| a_{1};\Theta;\ldots;a_{n} \right|+\ldots+\left| a_{1};a_{2};\ldots;\Theta\right|,$$

$$\left| a_{1};a_{2};\ldots;a_{n} \right|=Z^{n}\prod_{i=1}^{n} \psi_{t_{i}^{p}}, \text{and}$$

$$\left| a_{1};\ldots;\Theta;\ldots;a_{n} \right|=\left| \begin{matrix} Z\psi_{t_{2}^{p}} & \begin{matrix} \ldots& \theta\ldots\end{matrix} & 0 \\ \begin{matrix} 0 \\ \vdots\end{matrix} & \begin{matrix} \begin{matrix} \ldots\\ \vdots\end{matrix} & \begin{matrix} \theta\ldots\\ \vdots\ldots\end{matrix} \end{matrix} & \begin{matrix} 0 \\ \vdots\end{matrix} \\ 0 & \begin{matrix} \ldots& \theta\ldots\end{matrix} & Z\psi_{t_{n}^{p}} \end{matrix} \right|=\theta Z^{n-1}\prod_{j=1,j\neq i}^{n} \psi_{t_{i}^{p}}.$$

Hence, we can conclude that:

$$I\left( n \right)=Z^{n\frac{\alpha}{\beta}-1}\left[ Z\prod_{i=1}^{n} \psi_{t_{i}^{p}}+\theta\sum_{k\in K} \prod_{i\in k} \psi_{t_{i}^{p}} \right],\text{ where} K=\left\{ k\in2^{\left\{ 1,..,n \right\}} | \left| k \right|=n-1 \right\}.$$

Thus, the statement concerning the determinant *I*(*n*) has been proved. Moreover, it is obvious that all corner minors of the matrix $He\left( p,b_{p}^{*} \right)$ have the form of $I\left( 1 \right),..,I\left( \left| I_{3}^{p} \right| \right)$. The necessary condition that the function $W$reaches an optimum on $H_{p}$ (local or global) at the point $b_{p}^{*}\in{ReInt(H}_{p})$ is Condition (A2). The second order conditions that the function $W$reaches an optimum on $H_{p}$ (local or global) at the point $b_{p}^{*}\in{ReInt(H}_{p})$ are provided further. The conditions $Z>0, \theta<0 \text{and}\text{ }\psi_{t_{i}^{p}}<0, i=\bar{1,n}$, imply that $I\left( n \right)>0$ for all even values of *n* from 1 to $\left| I_{3}^{p} \right|$, and $I\left( n \right)<0$ for all odd values of *n* from 1 to $\left| I_{3}^{p} \right|$. It means that if the point $b_{p}^{*}\in{ReInt(H}_{p})$ satisfies Condition (A2), this point is the point of local maximum of the function$W$ on $H_{p}$. According to the first part of Proposition 1, $b_{p}^{*}$ is the unique point of global maximum of the function$W$ on $H_{p}$. Thus, this proposition is proved.

**Appendix B**

Consider the four following optimization problems (where *α*, *β*, *k*_1_, *k*_2_ and *C* > 0):

$$\left\{ \begin{aligned} W\left( b \right)=B^{\alpha}\left( b \right)V^{\beta}\left( b \right)\to{max}_{b}, \\ k_{1}B\left( b \right)+k_{2}V\left( b \right)\leq C, \\ b\in H. \end{aligned} \right.$$

(B1)

$$\left\{ \begin{aligned} W\left( b \right)=B^{\alpha}\left( b \right)V^{\beta}\left( b \right)\to{max}_{b}, \\ b\in H. \end{aligned} \right.$$

(B2)

$$\left\{ \begin{aligned} B\left( b \right)\to{min}_{b}, \\ k_{1}B\left( b \right)+k_{2}V\left( b \right)=C, \\ B\left( b \right)>\frac{C}{k_{1}\left( 1+\frac{\beta}{\alpha} \right)} , b\in H. \end{aligned} \right.$$

(B3)

$$\left\{ \begin{aligned} B\left( b \right)\to{max}_{b}, \\ k_{1}B\left( b \right)+k_{2}V\left( b \right)=C, \\ B\left( b \right)<\frac{C}{k_{1}\left( 1+\frac{\beta}{\alpha} \right)} ,b\in H. \end{aligned} \right.$$

(B3)’

Let $A\text{ be the set of all }b\in H\text{, such that B}\left( b \right)=\frac{C}{k_{1}\left( 1+\frac{\beta}{\alpha} \right)} \text{and } V\left( b \right)=\frac{C}{k_{2}\left( 1+\frac{\alpha}{\beta} \right)}.$

**Proposition 2**

There exist three cases that describe solutions of Problem (B1). They are as follows:

1. The resource constraint of Problem (B1) does not exclude some solutions of Problem (B2). Thus, these and only these points represent solutions of Problem (B1) (how to find these points, see Proposition 1 and Sections 3.2 – 3.3).
2. Assume that the resource constraint of Problem (B1) excludes solutions of Problem (B2) and the set *A* is non-empty. Then, *A* represents the set of all solutions of Problem (B1).
3. Assume that the resource constraint of Problem (B1) excludes solutions of Problem (B2) and the set *A* is empty. Assume that all trade-off functions are convex. First, we find solutions of Problems (B3) and (B3)’. Second, we use Proposition 1 the results of the second and third sub-sections of the section "Optimization model for the colony of cells of different types" to find the eligible (belonging to the domain of Problem (B1)) local maxima of Problem (B2). Solutions of Problem (B1) belong to the set of solutions of Problems (B3), (B3)’ and (B2) mentioned above. Assume that all trade-off functions are strictly concave or linear. Then, solutions of Problem (B1) belong to the set of solutions of Problems (B3) and (B3)’.

**Proof**

1. The resource constraint of Problem (B1) does not exclude some solutions of Problem (B2). Let *S* be the set of all solutions of Problem (B2), such that all points from *S* belong to the domain of Problem (B1). Let *b*^*^ be a solution belonging to *S*. It means that *W*(*b*^*^) ≥ *W*(*b*) for all *b* from *H*. Because the domain of Problem (B1) is a subset of *H*, for all *b* from the domain of Problem (B1), the following inequality holds *W*(*b*^*^) ≥ *W*(*b*), i.e., *b*^*^ represents a solution of Problem (B1). Conversely, let *b*^*^ be a solution of Problem (B1). Because *b*^*^ is a solution of Problem (B1), *S* is the non-empty set of all solutions of Problem (B2), such that all points from *S* belong to the domain of Problem (B1). So, we have *W*(*b*^*^) ≥ *W*(*b^#^*) ≥ *W*(*b*) for all *b*^#^ from *S* and for all *b* from *H*. It means that *b*^*^ is a solution of Problem (B1), i.e., *b*^*^ belongs to *S*.

2. Assume that the resource constraint of Problem (B1) excludes solutions of Problem (B2) and the set *A* is non-empty. Consider any point *b*^*^ from this set. We need to prove that *W*(*b*^*^) = *W*(*b***^’^**), for any other *b***^’^** from the set *A* and *W*(*b*^*^) > *W*(*b*), for any *b* that belongs to the domain of Problem (B1), but does not belong to *A*.

First, consider the following optimization problem:

$$\left\{ \begin{aligned} W\left( b \right)=B^{\alpha}\left( b \right)V^{\beta}\left( b \right)\to{max}_{b}, \\ k_{1}B\left( b \right)+k_{2}V\left( b \right)=C , \\ b\in H. \end{aligned} \right.$$

We can rewrite it as follows:

$$\left\{ \begin{aligned} W\left( b \right)=\frac{1}{k_{2}^{\beta}}B^{\alpha}\left( b \right)\left( C-k_{1}B\left( b \right) \right)^{\beta}\to{max}_{b}, \\ k_{1}B\left( b \right)+k_{2}V\left( b \right)=C , \\ b\in H. \end{aligned} \right.$$

(B4)

Let us study the behavior of the function $Z\left( B \right)=\frac{1}{k_{2}^{\beta}}B^{\alpha}\left( C-k_{1}B \right)^{\beta}$ on the set $B\in[0,\frac{C}{k_{1}}]$. We choose this closed interval, because for each point *b* from the domain of Problem (B4), *B*(*b*) belongs to this closed interval. Using standard optimization techniques, we determine that the function $Z\left( B \right)$ has only one stationary point $B^{*}=\frac{C}{k_{1}\left( 1+\frac{\beta}{\alpha} \right)}$, such that this stationary point belongs to the open interval $\left( 0,\frac{C}{k_{1}} \right)$. Furthermore, note that $Z\left( B^{*} \right)=\left( \frac{\alpha}{k_{1}} \right)^{\alpha}\left( \frac{\beta}{k_{2}} \right)^{\beta}\left( \frac{C}{\alpha+\beta} \right)^{\alpha+\beta}>0$ and $Z\left( 0 \right)=Z\left( \frac{C}{k_{1}} \right)=0$. It means that the function *Z*(*B*) reaches the maximum on the set $B\in[0,\frac{C}{k_{1}}]$ in the point $B^{*}=\frac{C}{k_{1}\left( 1+\frac{\beta}{\alpha} \right)}$ [4]. In other words, $Z\left( B^{*} \right)>Z\left( B \right) \text{for all }B\in[0,\frac{C}{k_{1}}]$.

Note that $B\left( b^{*} \right)=B\left( b\boldsymbol{'} \right)=B^{*}. \text{It means that }W\left( b^{*} \right)=W\left( b^{'} \right)=Z\left( B^{*} \right)$. Consider any point *b* that belongs to the domain of Problem (B1), but does not belong to *A*. There can be two cases. In the first case, *b* belongs to the domain of Problem (B4), but does not belong to *A*. It means that $B\left( b \right)\in\left[ 0,\frac{C}{k_{1}} \right]\backslash\left\{ B^{*} \right\}. \text{Consequently, we have determined that }W\left( b^{*} \right)=Z\left( B^{*} \right)>Z\left( B\left( b \right) \right)=W(b)$. In the second case, *b* does not belong to the domain of Problem (B4). It means that there exists some *σ*, 0 ≤ *σ* < *C*, such that the point *b* belongs to the domain of the following optimization problem:

$$\left\{ \begin{aligned} W\left( b \right)=B^{\alpha}\left( b \right)V^{\beta}\left( b \right)\to{max}_{b}, \\ k_{1}B\left( b \right)+k_{2}V\left( b \right)=\sigma, \\ b\in H. \end{aligned} \right.$$

(B5)

For this problem we know that $W\left( b \right)\leq\left( \frac{\alpha}{k_{1}} \right)^{\alpha}\left( \frac{\beta}{k_{2}} \right)^{\beta}\left( \frac{\sigma}{\alpha+\beta} \right)^{\alpha+\beta}<\left( \frac{\alpha}{k_{1}} \right)^{\alpha}\left( \frac{\beta}{k_{2}} \right)^{\beta}\left( \frac{C}{\alpha+\beta} \right)^{\alpha+\beta}=W\left( b^{*} \right)$. Thus, we have proved that if the set *A* is non-empty, then *A* represents the set of all solutions of Problem (B1).

3. Assume that the set *A* is empty and the resource constraint excludes solutions of Problem (B2). In this case our task is two-fold: (1) to examine the function *W* on the surface of the resource constraint, and (2) on the set of faces of the hyperparallelepiped *H* belonging to the domain of Problem (B1). In other words, it means that if $b^{*}$ is a solution of Problem (B1), then: (1) $b^{*}$ is a solution of Problem (B4) or (2) $b^{*}$ is a feasible point of local maximum of the function *W* on the set *H*. To solve the second problem, we can use the procedure presented in Appendix A (Proposition 1). To solve the first problem, consider Problem (B4). Let $b^{*}$ be the solution of Problem (B4). Introduce *L* – the set of all $B\in R$ such that there exists *b* that belongs to the domain of Problem (B4) and *B*(*b*) = *B*. Note that $L\subset\left[ 0,\frac{C}{k_{1}} \right]\backslash\left\{ \frac{C}{k_{1}\left( 1+\frac{\beta}{\alpha} \right)} \right\}$. Also, the set *L* is a non-empty and compact. Because the domain of Problem (B4) is compact, the function $B\left( b \right)=\sum_{i=1}^{N} b_{i}$ is linear (i.e., continuous function) and$B\left( b^{*} \right)\in L$. Consider the function$Z\left( B \right)=\frac{1}{k_{2}^{\beta}}B^{\alpha}\left( C-k_{1}B \right)^{\beta}$. Problem (B4) is equivalent to the maximization problem of the function $Z\left( B \right)$ on the set *L*. We have shown that the function $Z\left( B \right)$ reaches the maximum on the set $\left[ 0,\frac{C}{k_{1}} \right]$ in the point${B(b}^{*})$. Moreover, $Z\left( B \right)$ is monotonically increasing on the set $\left( 0,\frac{C}{k_{1}\left( 1+\frac{\beta}{\alpha} \right)} \right)$ and monotonically decreasing on the set$\left( \frac{C}{k_{1}\left( 1+\frac{\beta}{\alpha} \right)},\frac{C}{k_{1}} \right)$. Consequently, maximal value of the function $Z\left( B \right)$ on the set *L* can be attained in one of two points. The first one is the solution of Problem $B{\to max}_{B}, s.t. B\in L, B<\frac{C}{k_{1}\left( 1+\frac{\beta}{\alpha} \right)}$. The second one is the solution of Problem$B{\to min}_{B}, s.t. B\in L, B>\frac{C}{k_{1}\left( 1+\frac{\beta}{\alpha} \right)}$. But the first problem is equivalent to Problem (B3) and the second to Problem (B3)’. Finally, we can conclude that the solution of Problem (B4) belongs to the set of solutions of Problems (B3) and (B3)’.

Assume that all trade-off functions are strictly concave. In Appendix A we have shown that there is the unique point of local maximum of the function *W* on the set *H*, and this point represents the point of global maximum of the function *W* on the set *H*, i.e., the solution of Problem (B2). Since the resource constraint of Problem (B1) excludes solutions of Problem (B2), there are no feasible points of local maximum of the function *W* on the set *H*. Consequently, solutions of Problem (B1) belong to the set of solutions of Problems (B3) and (B3)’. Assume that all trade-off functions are linear. It is easy to show that the following statement is true: consider two points *b*^'^ and *b*^''^ from *H*, such that *b*^'^ ≠ *b*^''^, *B*(*b*^'^) ≠ *B*(*b*^''^) and *W*(*b*^'^) = *W*(*b*^''^) > 0. Then, for all ξ from (0, 1) the following inequality holds: *W*(ξ*b*^'^ + (1- ξ) *b*^''^) > *W*(*b*^'^). Using this statement and the arguments from Proposition 1, we can show that if *b*^1^ and *b*^2^ are points of local maximum of the function *W* on the set *H*, then *W*(ξ *b*^1^ + (1- ξ) *b*^2^) = *W*(*b*^1^) for all ξ from [0, 1] and points ξ*b*^1^ + (1- ξ) *b*^2^, where ξ belongs to [0, 1], are points of global maximum of the function *W* on the set *H*. Since the resource constraint of Problem (B1) excludes solutions of Problem (B2), there are no feasible points of local maximum of the function *W* on the set *H*. Consequently, the set of solutions of Problem (B1) belong to the set of solutions of Problems (B3) and (B3)’. Thus, Proposition 2 is proved.

**Appendix С**

Consider the following optimization problems, where *α*, *β*, *k*_1_, *k*_2_ and *C* > 0:

$$\left\{ \begin{aligned} {(-1)}^{y}B\left( b \right)\to{max}_{b,v}, \\ k_{1}B\left( b \right)+k_{2}V\left( v \right)=C, \\ {(-1)}^{y}B\left( b \right)<{(-1)}^{y}\frac{C}{k_{1}\left( 1+\frac{\beta}{\alpha} \right)} , \\ 0\leq v_{i}\leq\varphi_{i}\left( b_{i} \right),b_{i}\geq0,i=1,..,N. \end{aligned} \right.$$

(С*_y_*, *y* = 1, 2)

**Proposition 3**

There exist three cases that describe solutions of Problem (6) with trade-offs in the form of inequalities. They are as follows:

1. The resource constraint of Problem (6) with trade-offs in the form of inequalities does not exclude some solutions of the corresponding Problem (B2). These and only these points are solutions of Problem (6) with trade-offs in the form of inequalities.
2. Assume that the resource constraint in Problem (6) with trade-offs in the form of inequalities excludes all solutions of Problem (B2) and the set *A*' is non-empty. Then, all points of this set (and only these points) are solutions of Problem (6) with trade-offs in the form of inequalities.
3. Assume that the resource constraint in Problem (6) with trade-offs in the form of inequalities excludes all solutions of Problem (B2) and the set *A*' is empty. Assume that all trade-off functions are convex. First, we find solutions of Problems (C_1_) and (C_2_). Second, we use Proposition 1 to find eligible local maxima of Problem (B2). Solutions of Problem (6) with trade-offs in the form of inequalities belong to the set of solutions of Problems (C_1_), (C_2_) and local maxima of (B2) mentioned above. Assume that all trade-off functions are strictly concave or linear. Then, solutions of Problem (6) with trade-offs in the form of inequalities belong to the set of solutions of Problems (C_1_) and (C_2_).

The proof of Proposition 3 (not presented here for the sake of brevity) follows strictly the same arguments that have been used to prove Propositions 1 and 2 above.

## References

1. Rudin W. Principles of Mathematical Analysis. 3nd ed. Springer-Verlag; 1976.

2. Lang S. Calculus of Several Variables. 3nd ed. Springer-Verlag; 1987.

3. Chong EZ. An introduction to optimization, 2nd edition. Wiley-Interscience series in discrete mathematics and optimization; 2004.

4. Binmore KG. Calculus. Cambridge University Press; 1986
